# MERS-CoV ORF4b employs an unusual binding mechanism to target IMPα and block innate immunity

**DOI:** 10.1038/s41467-022-28851-2

**Published:** 2022-03-25

**Authors:** Thilini S. Munasinghe, Megan R. Edwards, Sofiya Tsimbalyuk, Olivia A. Vogel, Kate M. Smith, Murray Stewart, Justin K. Foster, Loretta A. Bosence, David Aragão, Justin A. Roby, Christopher F. Basler, Jade K. Forwood

**Affiliations:** 1 School of Biomedical Sciences, Charles Sturt University, Wagga Wagga, NSW 2678 Australia; 2 Center for Microbial Pathogenesis, Institute for Biomedical Sciences, Georgia State University, Atlanta, GA 30303 USA; 3 Paul Scherrer Institut, Swiss Light Source, 5232 Villigen PSI, Switzerland; 4 MRC Laboratory of Molecular Biology, Francis Crick Ave., Cambridge Biomedical Campus, Cambridge, CB2 0QH UK; 5 Diamond Light Source, Harwell Science and Innovation Campus, Didcot, OX11 0DE UK; 6 Department of Microbiology, Icahn School of Medicine at Mount Sinai, New York, NY 10029 USA

**Keywords:** X-ray crystallography, Viral infection, Virus-host interactions

## Abstract

The MERS coronavirus (MERS-CoV) is a highly pathogenic, emerging virus that produces accessory proteins to antagonize the host innate immune response. The MERS-CoV ORF4b protein has been shown to bind preferentially to the nuclear import adapter IMPα3 in infected cells, thereby inhibiting NF-κB-dependent innate immune responses. Here, we report high-resolution structures of ORF4b bound to two distinct IMPα family members. Each exhibit highly similar binding mechanisms that, in both cases, lack a prototypical Lys bound at their P2 site. Mutations within the NLS region dramatically alter the mechanism of binding, which reverts to the canonical P2 Lys binding mechanism. Mutational studies confirm that the novel binding mechanism is important for its nuclear import, IMPα interaction, and inhibition of innate immune signaling pathways. In parallel, we determined structures of the nuclear binding domain of NF-κB component p50 bound to both IMPα2 and α3, demonstrating that p50 overlaps with the ORF4b binding sites, suggesting a basis for inhibition. Our results provide a detailed structural basis that explains how a virus can target the IMPα nuclear import adapter to impair immunity, and illustrate how small mutations in ORF4b, like those found in closely related coronaviruses such as HKU5, change the IMPα binding mechanism.

## Abstract (web summary)

MERS-CoV ORF4b antagonizes host innate immune response, partially via blocking nuclear import adapter IMPα activity and preventing nuclear translocation of NF-κB. Here, Munasinghe and Edwards et al. biochemically and structurally define the interaction between ORF4b and IMPα-family members and find a non-canonical interaction between ORF4b NLS and IMPα2 and IMPα3.

## Introduction

The Middle East respiratory syndrome-related coronavirus (MERS-CoV) is a single-stranded positive-sense RNA virus that is closely related to other important human-infecting coronaviruses including severe acute respiratory syndrome-related coronavirus-1 (SARS-CoV-1) and SARS-CoV-2^1^. MERS-CoV is a highly pathogenic zoonotic agent that belongs to the subgenus *Merbecovirus* of the genus *Betacoronavirus*^2^. It emerged in Saudi Arabia in 2012 and has caused 2494 infections and 858 deaths to date^3^. MERS-CoV has demonstrated a capacity to quickly spread to new geographic areas, causing a major outbreak in South Korea in 2015^4^. Although MERS-CoV has thus far proven less transmissible between people than SARS-CoV-2, it has a markedly higher case fatality rate^5,6^, and so remains a concerning latent pandemic threat. Consequently, generating an improved understanding of the MERS-CoV-host interactions that drive severe pathological outcomes remains a critical prerequisite to understand the disease and to develop novel therapeutic strategies.

**Fig. 1:**
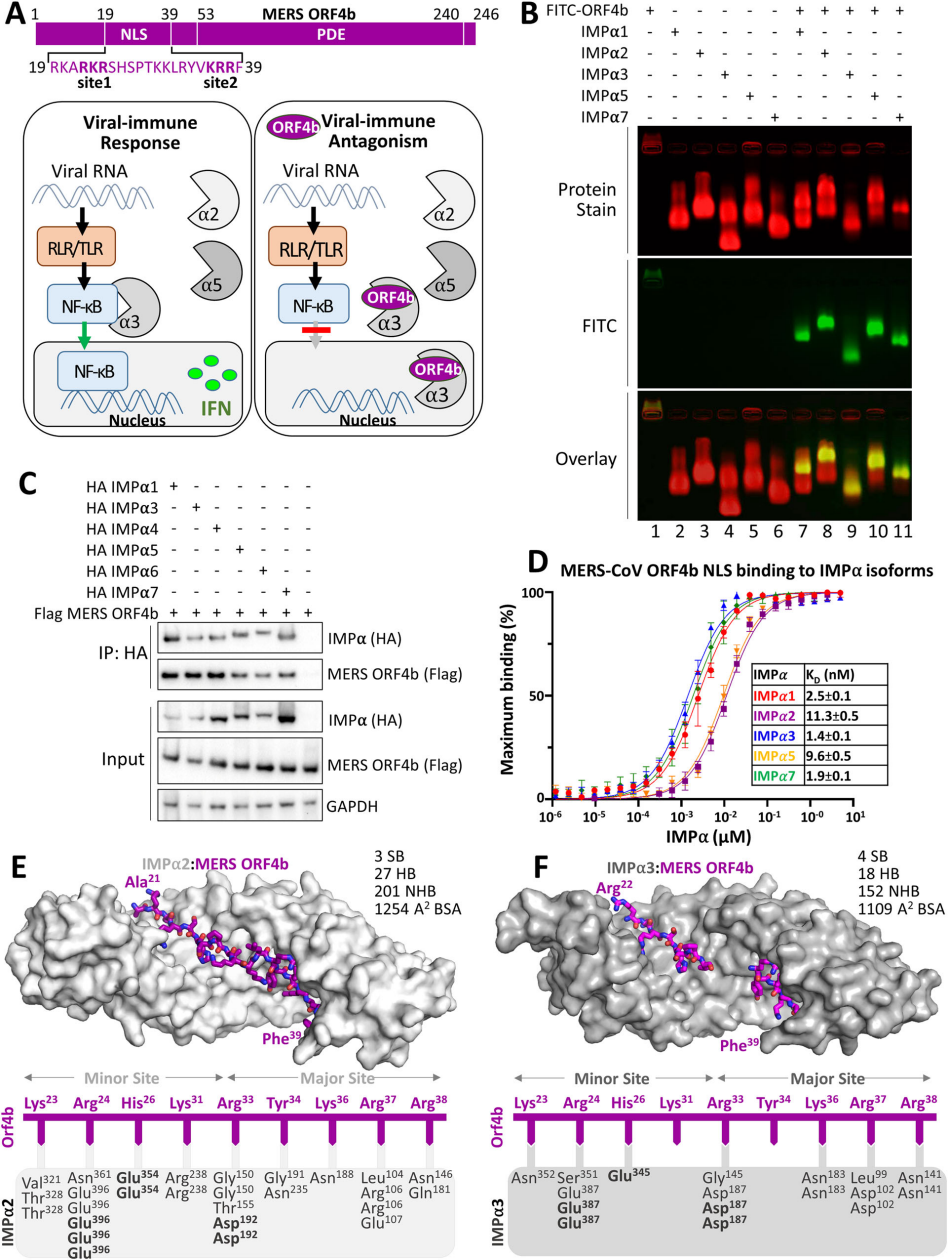
MERS ORF4b binding to IMPα is not isoform specific. **A** Schematic overview of the ORF4b protein. The region responsible for nuclear localization is contained with the N-terminus and spans two sites within residues 19-39. This region has been shown to be required for ORF4b to block the nuclear translocation of the p65 subunit of NF-ĸB, thereby blocking NF-ĸB-dependent cytokine production, by binding specifically to IMPα3. **B** Electro-mobility shift assay (EMSA) showing MERS ORF4b NLS binds with ΔIBB-IMPα isoforms spanning members from each of the three subfamilies (SF1: IMPα1/2; SF2: IMPα3; SF3: IMPα5/7). The MERS ORF4b NLS region peptide spans residues 19–39 and contains a FITC and Ahx linker (middle panel). Proteins were stained using Coomassie blue stain (top panel; red), and the overlay is represented in the bottom panel, where the complex produces a yellow color. **C** Co-immunoprecipitation (coIP) assay against HA-tagged IMPα isoforms and probed for the presence of full-length FLAG-tagged MERS-CoV ORF4b. GAPDH was used as an internal control to ensure consistent quantity of cell extracts were used in each assay. CoIP was repeated twice independently with similar results. **D** Fluorescence polarization assay measuring the direct binding between MERS-CoV ORF4b NLS and respective IMPα isoforms. Strong binding in the 1–10 nM range was observed across all IMPα isoforms. Each family member was able to bind in the low nM range (SF1: IMPα1 = 2.5 nM; SF2: IMPα3 = 1.4 nM; SF3: IMPα7 = 1.9 nM). Source data are provided as a Source Data file. *n* = 3 biologically independent experiments. Error bars represent mean values ± SEM. **E** Crystal structure of MERS-CoV ORF4b NLS bound to IMPα2 at 2.1 Å resolution. The IMPα is shown in light gray and surface mode, and the ORF4b shown in purple in stick mode. Pymol software was used to generate images^45^. A cartoon representation is shown below highlighting the hydrogen bonds and salt bridges (in bold). HB number of hydrogen bonds, SB number of salt bridges, NHB number of non-hydrogen bonds generated in PDBsum, BSA buried surface area calculated in PISA. **F** Crystal structure of MERS-CoV ORF4b NLS bound to IMPα3 at 2.5 Å resolution. The IMPα is shown in dark gray, and all other parameters are as per 1E. NLS nuclear localization signal, PDE phosphodiesterase domain, MERS Middle East respiratory syndrome-related coronavirus, IMPα importin alpha, α importin alpha, RLR RIG-I-like receptor, TLR Toll-like receptor, NF-κB Nuclear factor kappa light chain enhancer of activated B cells, IFN interferon, GAPDH Glyceraldehyde 3-phosphate dehydrogenase, HA haemagglutinin epitope tag, FITC fluorescein isothiocyanate.

The MERS-CoV ORF4b protein is a viral accessory protein that functions as an innate immune inhibitor (Fig. 1A) and strongly localizes to the nucleus, despite virus replication occurring exclusively within the cytoplasmic compartment^7^. This nuclear localization distinguishes ORF4b from other phosphodiesterase (PDE) proteins encoded by RNA viruses, such as the NS2 protein of betacoronaviruses within the subgenus *Embecovirus*, and VP3 of group A rotaviruses; both of which lack an NLS motif^8–10^. Interestingly the mammalian host PDE-domain containing A-kinase anchoring protein 7 gamma (AKAP7γ) (which has been proposed as the common genetic origin of ORF4b, NS2, and VP3^8,9^) does contain an N-terminal NLS motif and localizes strongly to the nucleus^8^, however, the NLS region is not homologous to that of merbecovirus ORF4b. ORF4b nuclear localization has been attributed to two short clusters of basic residues (nuclear localization signal sites NLS1 and NLS2) in the ORF4b N-terminal domain, while the C-terminus harbors a PDE domain (Fig. 1A)^7,10^. The innate immune inhibition functions attributed to ORF4b include inhibition of interferon beta (IFNβ) and IFN lambda (IFNλ) production, as well as inhibition of cytokine production that is mediated by the NF-ĸB p50/p65 heterodimer^7,11–14^. ORF4b PDE activity inhibits the innate antiviral 2′-5′ oligoadenylate (2–5 A) synthetase (OAS)-RNase L pathway by degrading 2–5 A produced by OAS, thus preventing activation of the antiviral enzyme RNase L via the 2–5A second messenger molecule^10,12^.

Although several studies demonstrated that ORF4b contributes to innate immune inhibition^7,10–12,15^ the mechanisms by which the NLSs facilitate such functions are incompletely understood. One notable study found that, in the context of MERS-CoV infection, ORF4b blocks the nuclear translocation of the p65 subunit of the NF-ĸB heterodimer, thereby blocking NF-ĸB-dependent cytokine production^11^. This was attributed to ORF4b binding preferentially (via its NLS sequences) to the host nuclear import protein IMPα3 (also known as KPNA4), which is responsible for the nuclear translocation of NF-κB. Consistent with this hypothesis, wild-type ORF4b, but not ORF4b NLS mutants, outcompete p65 for IMPα3 binding in immunoprecipitation assays^11^. These data place ORF4b among several viral proteins that reportedly block innate immune signaling by disrupting IMPα interactions with key host antiviral transcription factors^16–18^. Other examples include Ebola virus VP24^16,19^, hantavirus nucleoprotein^17^, and Japanese encephalitis virus NS5^18^.

The structural basis for the ORF4b:IMPα interaction and the competition with other IMPα cargo are important prerequisites for understanding how viral proteins can interfere with IMPα nuclear import functions to suppress innate immunity. Additionally, investigating functional variation within NLS regions among MERS-CoV and related merbecoviruses (including the bat merbecovirus HKU5) has the potential to identify altered interactions with nuclear import receptors with important consequences for host range, virulence and pathogenesis^7^. Here we provide a detailed, structural basis for the interaction between MERS-CoV ORF4b and its innate immune suppression target, the nuclear import adapter IMPα. We show that ORF4b binds to both the major and minor NLS binding sites on the IMPαs through a novel and highly unusual way that is conserved across two IMPα family members. Through structure-guided mutational analysis, we show that this interface is important for MERS ORF4b-mediated inhibition of innate immune signaling. Structural analysis of the NF-κB component p50 bound to IMPα2 and IMPα3 demonstrates that there is a direct overlap between the binding sites for this host transcription factor component and its competitive ORF4b inhibitor. Finally, we observe that, despite harboring conserved elements within these NLS regions, the precise way in which IMPαs interact with ORF4b from related viruses cannot be predicted from sequence alone, with distinct IMPα binding mechanisms existing for MERS-CoV related merbecoviruses including HKU5.

## Results

### MERS-CoV ORF4b bind IMPα subfamily members with high affinity

MERS-CoV ORF4b plays an important role in innate immune inhibition, where it is believed to block NF-ĸB-dependent cytokine production through preferential binding to the host nuclear import protein IMPα3 via its NLS sequences (sites 1 and 2 spanning residues 19–39; Fig. 1A)^11^. Understanding the mechanisms through which cargo binds specifically to IMPα isoforms is complicated by the high structural and sequence similarity across IMPα isoforms (particularly in the cargo binding regions) and has only been described recently for a small number of cargo^20^. Because MERS-CoV ORF4b has been reported to bind specifically to IMPα3, we sought to analyze the interaction interface using a combination of biophysical and structural approaches. Using recombinantly expressed IMPα isoforms spanning members from each of the three subfamilies (SF1: IMPα1/2; SF2: IMPα3; SF3: IMPα5/7), agarose gel mobility shift assays were performed in the absence and presence of a FITC-tagged MERS-CoV ORF4b peptide spanning residues 19–39 (henceforth simply referred to as “NLS”). In the absence of binding partners, the FITC-tagged peptide migrated towards the cathode (Fig. 1B, lane 1), whereas the IMPα proteins migrated towards the anode (Fig. 1B, lanes 2–6). In the presence of IMPα isoforms, the FITC-tagged peptide shifted migration towards the anode, forming a distinct band that migrated differently to both IMPα-only and peptide-only samples. These results suggest that the FITC-tagged ORF4b NLS peptide was capable of binding isoforms from each of the three IMPα subfamilies. To compare this result within a human cell expression context, we performed co-immunoprecipitation assays against respective full-length, HA-tagged IMPα isoforms, and probed for the presence of full-length FLAG-tagged MERS-CoV ORF4b. Consistent with the gel mobility shift assays, we found that MERS-CoV ORF4b bound to all IMPα isoforms tested (Fig. 1C and Supplementary Fig. 1). Next, we quantitatively measured the direct binding interaction with MERS-CoV ORF4b NLS to respective IMPα isoforms using fluorescence polarization. We observed very strong binding in the low nM range across all IMPα isoforms (Fig. 1D SF1: IMPα1, 2.5 nM; SF2: IMPα3, 1.4 nM; SF3: IMPα7, 1.9 nM). Indeed, the slight differences in the low nanomolar binding affinities of MERS-CoV ORF4b NLS to IMPα1, IMPα3, and IMPα7 were determined not to be statistically significant (one-way ANOVA followed by Tukey’s test, *p* > 0.1); therefore, interactions with the NLS region of this viral factor were equally strong across members of the three IMPα families. Because the results indicated that MERS-CoV ORF4b NLS was able to bind IMPα isoforms with high affinity and showed little preference for a specific isoform, we examined the molecular interface with two IMPα isoforms using high-resolution structural approaches. Specifically, we crystallized the MERS-CoV ORF4b NLS bound to both IMPα2 and IMPα3 and resolved the structures to 2.1 Å and 2.5 Å resolution respectively (see Supplementary Table 1 for data collection and refinement statistics). We identified consistent binding patterns, with MERS-CoV ORF4b residues Lys^23^, Arg^24^, His^26^, Arg^33^, Arg^37^, and Arg^38^ bound to both IMPα2 and IMPα3 at structurally equivalent interfaces (Fig. 1E, F) (see Supplementary Tables 2–3 for hydrogen bond interactions; Supplementary Fig. 2). In the MERS-CoV ORF4b:IMPα2 structure, we were able to resolve slightly more of the MERS-CoV ORF4b including the region Lys^30^ Lys^31^, Leu^32^ between the major and minor sites. There were also two additional ORF4b residues contributing to IMPα2 binding when compared to the IMPα3 structure (Lys^31^ and Tyr^34^), however, these did not appear to contribute significantly to the binding affinity (Fig. 1D). Overall, our structural and biochemical data confirm a direct and high-affinity interaction between MERS-CoV ORF4b and IMPα subfamilies without a marked preference between isoforms.

### MERS-CoV ORF4b interacts with IMPα2 and IMPα3 using a novel Arg P0 binding mechanism rather than the canonical Lys P2 at the major NLS binding site

**Fig. 2:**
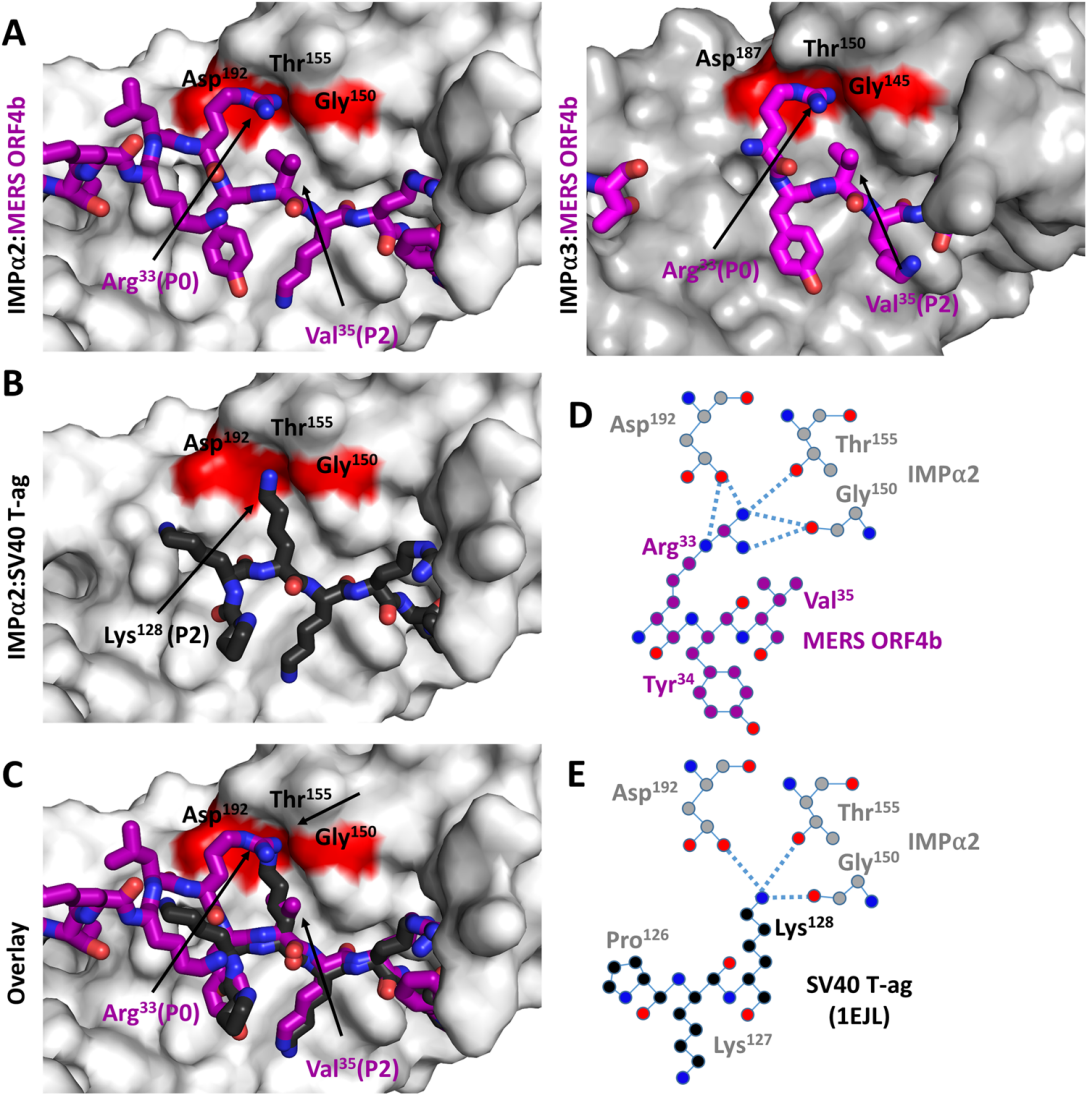
MERS ORF4b interacts with IMPα2 via an unusual P0 Arg binding mechanism at the major site. **A** Detailed view of MERS ORF4b bound at the major site of IMPα2 (left) and IMPα3 (right). IMPα2 and α3 are colored light and dark gray respectively and shown in surface view, and the MERS ORF4b is colored magenta and shown in stick view. Pymol software was used to generate images^45^. MERS-CoV ORF4b binds IMPα without the use of a Lys at the P2 position, and instead has a Val at this position. This is compensated by an Arg at the P0 position. **B** Comparison view of the well-characterized SV40 T-ag NLS bound to IMPα^24^, showing a well-characterized Lys at the P2 site. Other viral NLSs using this canonical Lys P2 site are displayed in Supplementary Fig. 2. **C** Overlay of MERS ORF4b and SV40 T-ag binding at the P2 site on IMPα2. **D** Detailed hydrogen bonding mediating the MERS ORF4b Arg^33^ P0 at the Gly, Thr, Asp bonding pocket of IMPα2. Bonds are denoted as dotted lines between atoms, and coloring is preserved within **A**. **E** Detailed hydrogen bonding mediating the SV40 T-ag Lys P2 at the Gly, Thr, Asp bonding pocket of IMPα2. Bonds are denoted as dotted lines between atoms, and coloring is preserved within **B**. MERS Middle East respiratory syndrome-related coronavirus, IMPα importin alpha, SV40 T-ag Simian virus 40 large T antigen.

Our high-resolution crystal structures showed that MERS-CoV ORF4b occupies an extensive interface across both IMPα2 and IMPα3. MERS-CoV ORF4b bound both the major NLS binding site, through residues Arg^33^, Lys^36^, Arg^37^, and Arg^38^, as well as the minor site through residues Lys^23^, Arg^24^, and His^26^, in both IMPα2 and IMPα3 (Fig. 1E, F). Strikingly, the binding interface of both structures lacked a Lys at the P2 position within the major site (Fig. 2A) that is seen with other NLSs. This was highly unusual because this site has been shown to contribute 50% of the binding energy at the major site, and a Lys at the P2 position is present in >95% of structures, including IMPα bound structures of Hendra and Nipah virus W proteins^21^, HIV Tat^22^, dengue and Zika virus NS5 proteins^23^, SV40 T-ag (Fig. 2B)^24^, and influenza A virus PB2^25^ (see also Supplementary Fig. 3). In the structures of MERS-CoV ORF4b bound to IMPα2 and IMPα3, this position is instead occupied by Val^35^ and neither its side chain nor main chain formed bonded interactions with IMPα2 or IMPα3. To compensate for this loss of binding, we observed that the side chain of Arg^33^, positioned two residues before where a P2 Lys would normally be, was bound at this pocket as can be seen in Fig. 2C that presents an overlay of ORF4b and SV40 T-ag NLS structures. This MERS-CoV ORF4b Arg P0 forms near identical bonding patterns to the canonical Lys P2 major site binding, with both forming H-bonds and salt bridge interactions with Gly^150^, Thr^155^, and Asp^192^ on IMPα2 (Fig. 2D, E), and the equivalent Gly^145^, Thr^150^, Asp^187^ interactions with IMPα3 (Fig. 2A right panel). In summary, MERS-CoV ORF4b interacts in a novel way with both IMPα2 and IMPα3.

### Mutations within the MERS-CoV ORF4b NLS alter its binding to IMPαs, switching to a canonical P2 Lys

**Fig. 3:**
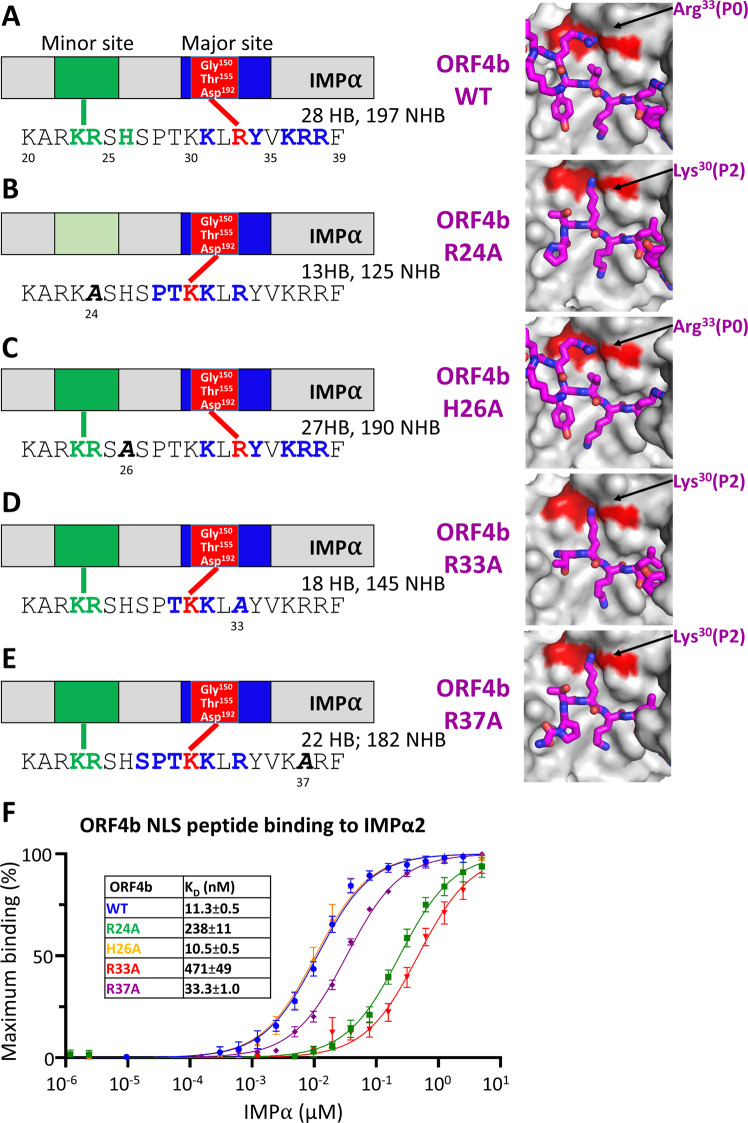
Mutations to key residues at the MERS ORF4b binding interface change the binding mechanism to canonical Lys P2 site. **A** A wild-type peptide of MERS ORF4b was used as a control and crystallized with IMPα2. As expected, the Arg33 bound at the Gly150, Thr155, and Asp192 pocket. A summary cartoon is presented, highlighting the minor and major site interactions and the structure presented in the right panel. A list of hydrogen-bonded interactions is presented in Supplementary Table 5. **B** An Arg24Ala mutation within the minor site, denoted in bold and italics with the numbering below the mutation is presented, showing a loss of minor site binding and change in binding mechanism at the major site. A list of hydrogen-bonded interactions is presented in Supplementary Table 6. **C** A His26Ala mutation, depicted as above, had little effect on the binding. A list of hydrogen-bonded interactions is presented in Supplementary Table 7. **D** An Arg33Ala mutation, depicted as above, caused a change in binding at the major site to the canonical P2 Lys binding. A list of hydrogen-bonded interactions is presented in Supplementary Table 8. **E** An Arg37Ala mutation, depicted as above, caused a change in binding at the major site to the canonical P2 Lys binding. A list of hydrogen-bonded interactions is presented in Supplementary Table 9. **F** Fluorescence polarization measuring the binding interaction of IMPα2 with the crystallized mutants (*n* = 3 biologically independent experiments). Error bars represent mean values ± SEM. R24A and R33A mutants had the greatest effect on binding. Source data are provided as a Source Data file. NLS nuclear localization signal, IMPα importin alpha, WT wild-type, HB number of hydrogen bonds, NHB number of non-hydrogen bonds.

To examine the effect of mutations within the key IMPα binding determinants of MERS-CoV ORF4b, we also crystallized MERS-CoV ORF4b wild-type (WT) and mutant NLS peptides in complex with IMPα2 (Fig. 3A–E; Supplementary Figs. 2 and 4; Supplementary Tables 4–5 for data collection and refinement statistics and Supplementary Tables 6–10 for hydrogen bond interactions). We found that, compared with WT (Fig. 3A), a MERS-CoV ORF4b mutation of Arg^24^ to Ala (R24A) bound only at the major site of IMPα2. Strikingly, with the lack of minor site binding, there was a distinctly different binding register at the major site (Fig. 3B). Here, the major site was occupied with a canonical Lys at the P2 position (MERS-CoV ORF4b Lys^30^), and indeed, the ^28^PTKKLR bound at the major site represents a very typical major site NLS. We next examined the effect of a MERS-CoV ORF4b His^26^ to Ala (H26A) mutation within the minor site and found that the structure was almost identical to the MERS-CoV WT ORF4b NLS (Fig. 3C). The structure of a MERS-CoV ORF4b Arg^33^ to Ala (R33A) mutant bound to IMPα2 showed a change in the major site binding register similar to that of R24A, with Lys^30^ positioned at the P2 site, with minor site binding retained (Fig. 3D). Finally, we examined the crystal structure of a MERS-CoV ORF4b Arg^37^ to Ala (R37A) mutant bound to IMPα2 and found that the major site was also disrupted and formed a Lys^30^ P2 register similar to the R24A and R33A mutants (Fig. 3E). Overall, Arg^24^ and Arg^33^ are key binding determinants within the minor and major sites respectively, with Arg^33^ mediating a novel P0 binding site interaction, whereas point mutations alter the binding register to a canonical P2 Lys binding mechanism. Possible implications for merbecovirus evolution are discussed below.

### Mutations to NLS binding sites reduce binding affinities across IMPα family members

**Fig. 4:**
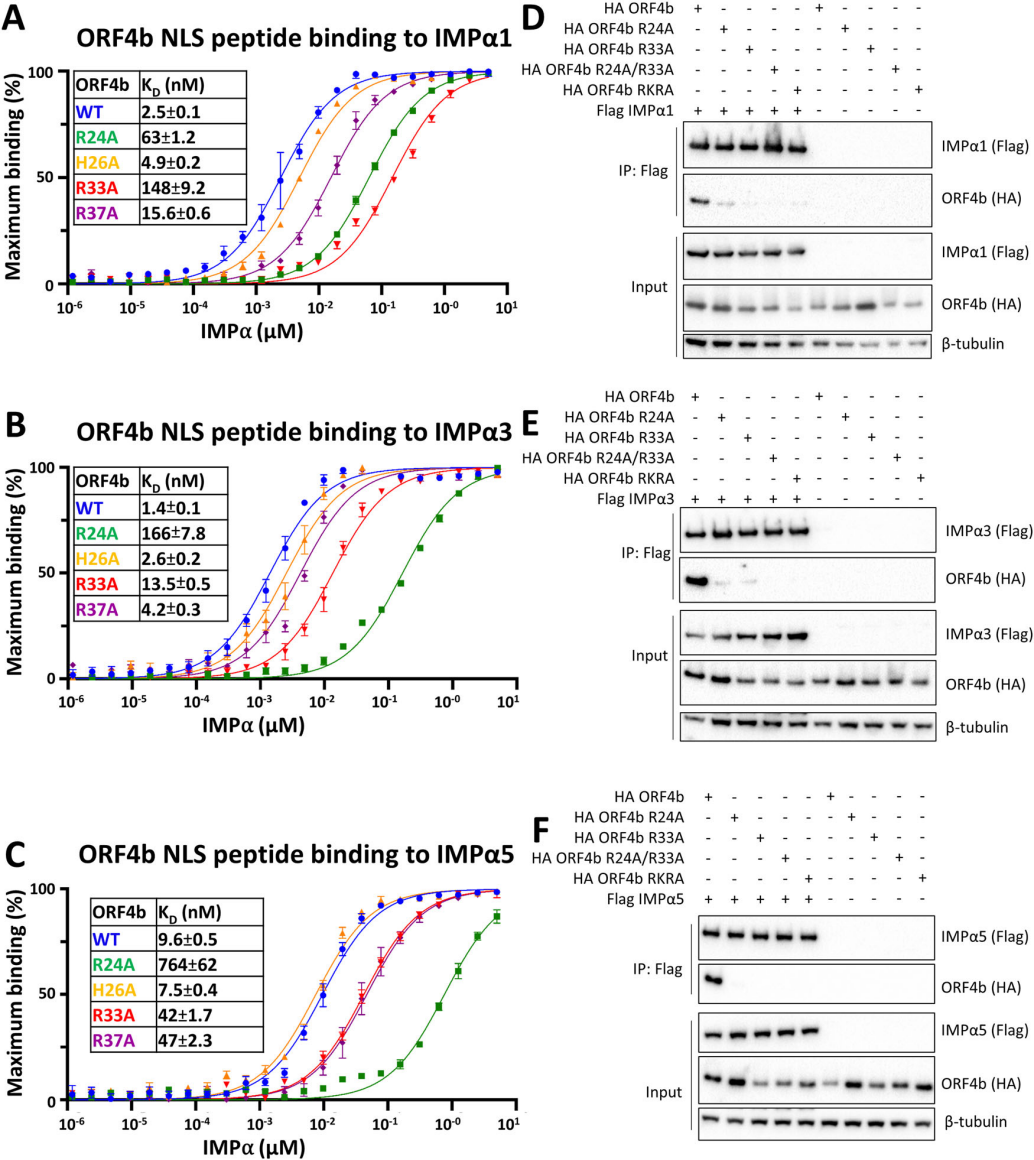
Mutations within key binding determinants at the MERS ORF4b binding interface alter binding efficiency to IMPα subfamilies. Fluorescence polarization measurements were undertaken to determine the affinity of interaction of MERS ORF4b and mutants across the IMPα family members SF1 (**A**), SF2 (**B**), SF3 (**C**). *n* = 3 biologically independent experiments and error bars represent mean values ± SEM for **A**–**C**. Source data are provided as a Source Data file. Respective immunoprecipitation assays were performed, measuring the interaction in the strongest mutants (R24A, R33A), and a control mutant RKRA (RKR24 to AAA24) in SF1 (**D**), SF2 (**E**), and SF3 (**F**). Assays were performed against HA-tagged IMPα isoforms and probed for the presence of full-length FLAG-tagged MERS-CoV ORF4b. β-tubulin was used as an internal control to ensure consistent quantity of cell extracts were used in each assay. CoIP’s were repeated twice for IMPα1 and α5 and three times for IMPα3. NLS nuclear localization signal, IMPα importin alpha, HA haemagglutinin epitope tag.

To further understand the effect of key MERS-CoV ORF4b binding determinants at the IMPα interface, we examined the strength of IMPα2 binding for FITC-tagged ORF4b wild-type (WT) and mutant NLS peptides using a fluorescence polarization assay (Fig. 3F). From this quantitative experiment, we determined that R24A at the minor site binding region and R33A at the major site binding region both reduced IMPα2 binding to the greatest extent, with binding affinities of 238 nM (significant ~20-fold reduction compared to WT; one-way ANOVA followed by Tukey’s test, *p* = 0.0002) and 471 nM (significant ~40-fold reduction compared to WT; one-way ANOVA followed by Tukey’s test, *p* < 0.0001), respectively. In contrast, the R37A mutation reduced IMPα2 binding to a smaller extent (33 nM; ~3-fold reduction, not significant), whereas H26A exhibited IMPα2 binding affinity that was effectively unchanged compared to WT (~10 nM, not significant). Because such highly similar binding patterns were seen for both IMPα2 and IMPα3, we also examined the effect of these interactions across multiple IMPα subfamilies including IMPα1, IMPα3, and IMPα5. We observed a similar trend to that observed with IMPα2, where mutations R24A and R33A resulted in large reductions in binding affinity (Fig. 4A–C). These interactions were also examined in a cellular context through immunoprecipitation assays, where we found that R24A and R33A, as well as a control mutant derived from the literature comprised of R22A/K23A/R24A (RKRA)^7,11^, reduced full-length ORF4b interaction across all IMPα subfamilies (Fig. 4D–F and Supplementary Figs. 5–7).

### MERS-CoV ORF4b mutations affect nuclear localization

**Fig. 5:**
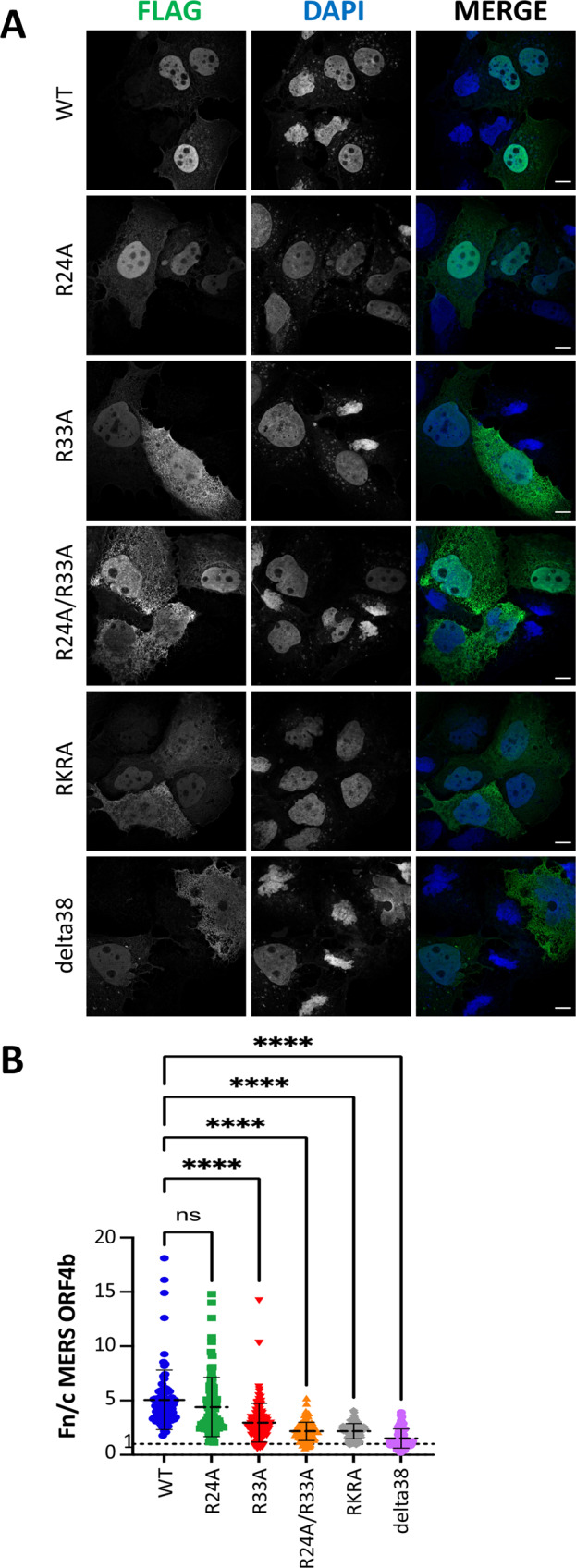
MERS ORF 4b protein localizes to the nucleus while mutations at the IMPα interface impair nuclear import efficiency. **A** Confocal microscopy images captured using a Zeiss LSM 800 confocal microscope of Huh7 cells transfected with Flag-tagged MERS ORF4b plasmid and stained using an anti-FLAG tag monoclonal antibody conjugated to Alexa Fluor 488. DAPI stain was used to identify the nuclear boundary within the cell, and merged panels represent an overlay of FLAG and DAPI panels. Significant reductions in nuclear import were observed for the R33A, R24A/R33A, RKR24/AAA24 (RKRA), and delta38 ORF4b constructs. The experiment was performed independently twice, with similar results. The scale bar is 10 μm. **B** Quantitation of the nuclear to cytoplasmic fluorescence signal (Fn/c) was measured in 88 cells for the wild-type protein, 85 cells for the R24A mutant protein; 139 cells for the R33A mutant protein; 118 cells for the R24A/R33A; 121 cells for the RKRA mutant protein; and 60 cells for the delta38 truncated protein, with error bars indicating mean values ± standard deviation. Statistical significance for the indicated comparisons was determined by a one-way ANOVA, followed by the Dunnett correction: ns no significant difference, ****p < 0.0001. Source data are provided as a Source Data file. MERS Middle East respiratory syndrome-related coronavirus, WT wild-type, DAPI 4′,6-diamidino-2-phenylindole, Fn/c fluorescence intensity in the nucleus divided by fluorescence intensity in the cytoplasm.

Based on our crystal structure and biochemical data indicating that Arg^24^ and Arg^33^ play key roles in binding to the minor and major sites respectively, we examined how these mutations altered nuclear localization of MERS-CoV ORF4b. We found that mutation of R24A alone had a small effect on the nuclear import of ORF4b, whereas mutation of R33A alone, or in combination with R24A (R24A/R33A), significantly decreased the nuclear localization of the mutant ORF4b proteins (Fig. 5A, B). The R24A/R33A double mutant demonstrated a loss of nuclear import similar to the previously described RKRA mutant (R22A/K23A/R24A)^7,11^ and an N-terminal deletion mutant (delta38) lacking the first 38 amino acids of ORF4b (Fig. 5A, B).

### NF-κB p50 and MERS-CoV ORF4b bind at overlapping sites on IMPαs

**Fig. 6:**
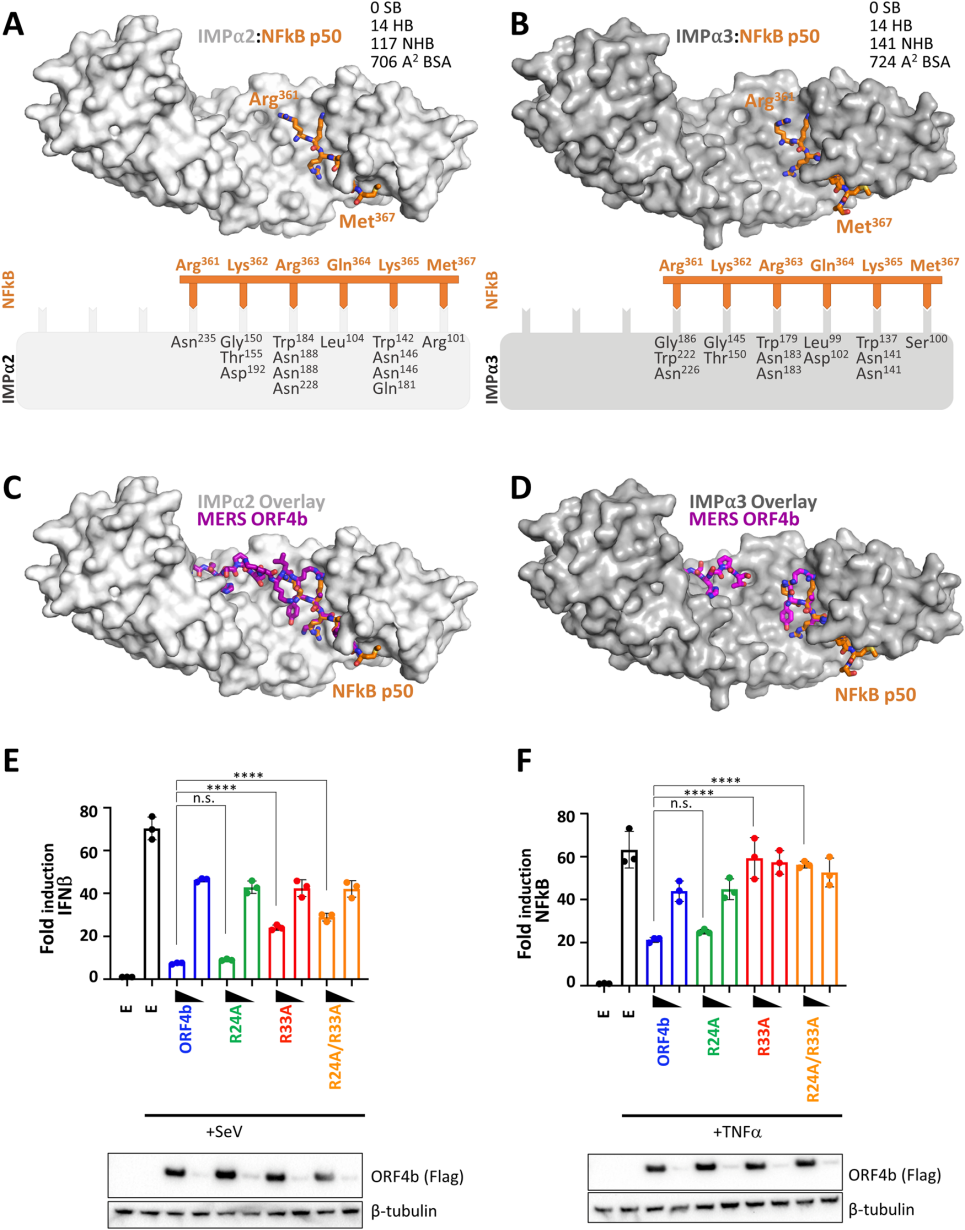
NF-ĸB p50 binding on IMPα2 and IMPα3 directly overlap with MERS ORF4b. **A** Crystal structure of NF-ĸB p50 bound to IMPα2 at 2.6 Å resolution (see Supplementary Table 11 for data collection and refinement statistics). IMPα2 is shown in light gray and surface mode, and the p50 is shown in orange in stick mode. Pymol software was used to generate images^45^. A cartoon representation is shown below highlighting the hydrogen bonds (see Supplementary Table 12 for a full list of hydrogen bond interactions). HB number of hydrogen bonds, SB number of salt bridges, NHB number of non-hydrogen bonds generated in PDBsum, BSA  buried surface area calculated in PISA. **B** Crystal structure of NF-ĸB p50 ORF4b NLS bound to IMPα3 at 2.15 Å resolution (see Supplementary Table 11 for data collection and refinement statistics). The IMPα3 is shown in dark gray and surface mode, and the p50 is shown in orange in stick mode. Pymol software was used to generate images^45^. A cartoon representation is shown below highlighting the hydrogen bonds (see Supplementary Table 13 for a full list of hydrogen bond interactions). **C** IMPα2 bound to ORF4b (as per Fig. 1E), and overlayed with the crystal structure of IMPα2 bound to p50 (as per A) showing a direct overlap at the major site. **D** IMPα3 bound to ORF4b (as per Fig. 1F), and overlayed with the crystal structure of IMPα3 bound to p50 (as per B) showing a direct overlap at the major site. **E** Reporter assay testing inhibition of SeV-induced activation of the IFNβ promoter by ORF4b and the indicated ORF4b mutants. Empty vector (E) served as a control and samples were infected with SeV as indicated. Error bars represent the mean values ± standard deviation for triplicate experiments. Statistical significance was determined by a one-way ANOVA followed by Tukey’s test; n.s. no significance; ****p < 0.0001. *n* = 3 biologically independent experiments. Source data are provided as a Source Data file. **F** Reporter assay testing inhibition of TNFα-induced activation of an NF-κB-responsive promoter by ORF4b and the indicated ORF4b mutants. Empty vector (E) served as a control and samples were treated with TNFα as indicated. Error bars represent the mean values + /− standard deviation for triplicate experiments. Statistical significance was determined by a one-way ANOVA followed by Tukey’s test; n.s. no significance; ****p < 0.0001. *n* = 3 biologically independent experiments. Source data are provided as a Source Data file. MERS Middle East respiratory syndrome-related coronavirus, IMPα importin alpha, NFkB Nuclear factor kappa light chain enhancer of activated B cells, IFNβ interferon β, SeV Sendai virus, TNFα Tumor necrosis factor α.

Previous reports indicated that MERS-CoV ORF4b can inhibit the innate immune response by blocking NF-κB signaling^11^. Since structural data of the binding site of NF-κB on IMPα remains elusive, we tested whether these sites overlapped. We crystallized the well-characterized NLS of p50^26^ in complex with both IMPα2 and IMPα3, and found that it bound at the major site of both IMPα2 and IMPα3 through highly similar interactions involving p50 residues ^361^RKRQKM (Fig. 6A, B) in which Lys^362^ bound at the P2 site (see Supplementary Fig. 4 and Supplementary Table 11 for data collection and refinement statistics, and Supplementary Tables 12 and 13 for hydrogen bond interactions). Overall, there was clear overlap in the binding sites of MERS-CoV ORF4b and NF-κB on both IMPα2 and IMPα3 (Fig. 6C, D). To examine whether the key binding determinants identified at the MERS-CoV ORF4b:IMPα interface could affect ORF4b inhibition of immune signaling, we tested the ORF4b R24A and R33A mutants for their ability to reduce IFNβ (SeV induction) and NF-κB (TNFα induction) responses. Consistent with the other data, we found that Arg^33^ was the most potent single point mutation in reducing the ability of ORF4b to inhibit immune effects in both HEK293T cells (Fig. 6E, F and Supplementary Figs. 8 and 9) and a human airway epithelial cell-derived cell line A549 (Supplementary Figs. 10 and 11). Because we were unable to obtain similar structural data for an IMPα:p65 complex, we examined the ability of MERS ORF4b to inhibit NF-κB p65 nuclear import during a TNFα response and found a marked reduction in nuclear import of this subunit. Consistent with the above data, the R33A mutant was significantly impaired in its inhibition of p65 nuclear import compared to WT ORF4b (Supplementary Figs. 12 and 13).

### Related merbecoviruses show different IMPα binding mechanisms

**Fig. 7:**
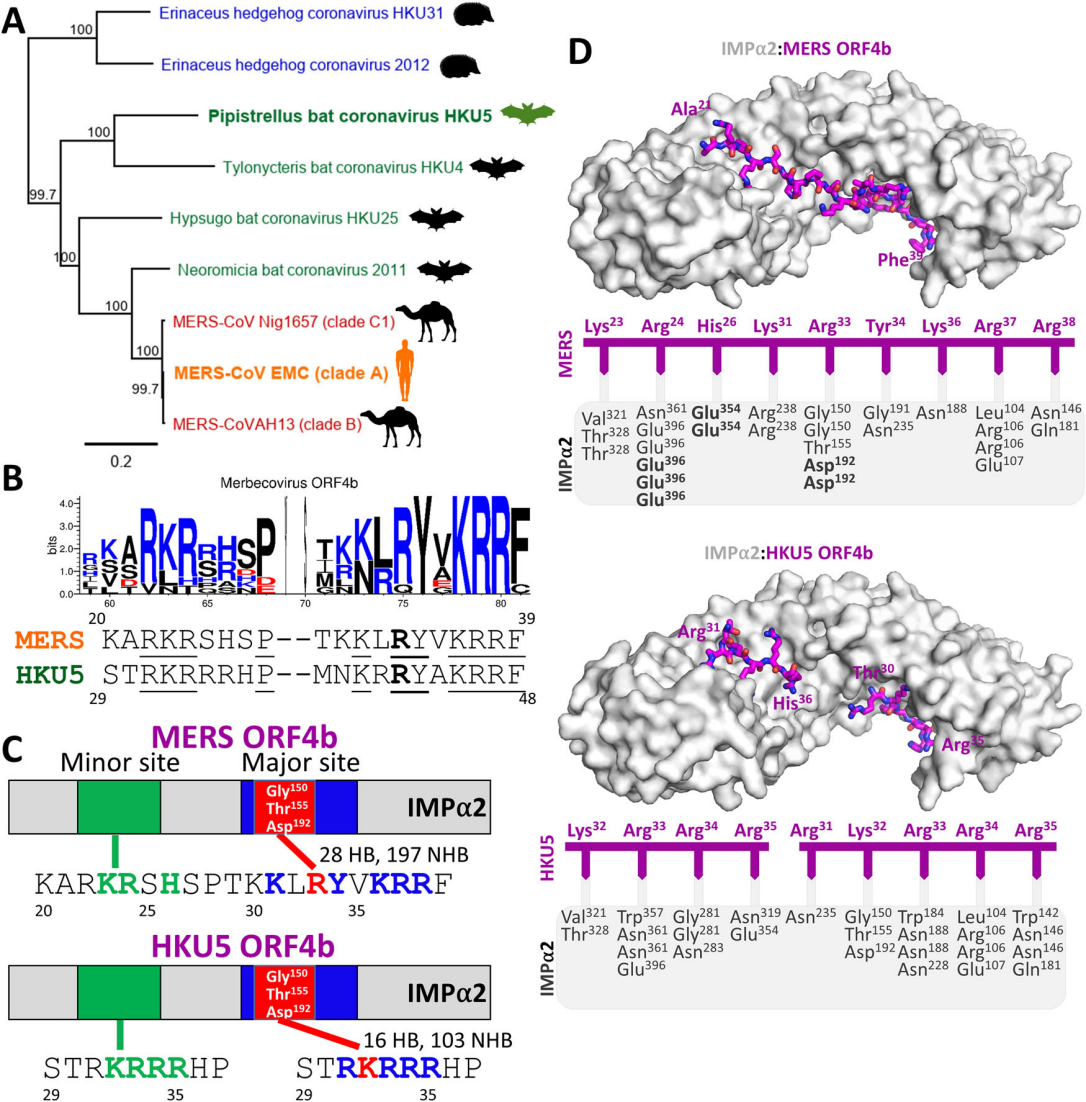
Related merbecovirus HKU5 binds IMPα through a different mechanism to MERS ORF4b. **A** Maximum likelihood tree of complete genome sequences of select viruses within subgenus *Merbecovirus*. Virus names are colored in accordance with the host from which the sequences were derived (blue = hedgehogs, green = bats, red = camels, and orange = humans). Numbers next to nodes indicate bootstrap proportion support, and the scale indicates the number of nucleotide substitutions per site. The Bat norbecovirus (HM211099.1) outgroup was used to root the tree. **B** Sequence logo demonstrating conservation of positively charged amino acids (blue) within the NLS region of ORF4b from the merbecoviruses in panel A (see Supplementary Fig. 10 for the full multiple sequence alignment). Below the sequence logo, the NLS region amino acids of MERS-CoV and HKU5 ORF4b are aligned. Conserved residues are underlined and the P0 Arg identified in MERS-CoV is in bold. **C** Cartoon representation highlighting the minor and major site interactions within MERS ORF4b and HKU5 ORF4b binding to IMPα2. **D** Crystal structure of HKU5 ORF4b bound to IMPα2 at 2.2 Å resolution (see Supplementary Table 11 for data collection and refinement statistics) and a comparison of MERS ORF4b bound to IMPα2 as per Fig. 1E. The IMPα is shown in light gray and surface mode, and the ORF4b shown in purple in stick mode. Pymol software was used to generate images^45^. A cartoon representation is shown below highlighting the hydrogen bonds and salt bridges (in bold) and the structure presented in the right panel. A list of hydrogen-bonded interactions is presented in Supplementary Table 14. MERS/MERS-CoV Middle East respiratory syndrome-related coronavirus, HKU5 Pipistrellus bat coronavirus HKU5, IMPα importin alpha.

Based on our observation that mutations within the MERS-CoV ORF4b dramatically changed the binding register on IMPα, we examined the conservation of the NLS binding region in related merbecoviruses (Fig. 7A). Because HKU5 ORF4b had been studied previously and shown to traffic to the nucleus^7^, and an analysis of the two NLS domains between the two viruses revealed only minor differences (Fig. 7B and Supplementary Fig. 14), we examined how differences could potentially result in changes in the mode of IMPα binding. Accordingly, we crystallized IMPα2 in complex with HKU5 ORF4b and found that, rather than utilizing the Arg/Val binding register motif of MERS-CoV ORF4b, HKU5 ORF4b bound IMPα2 using the canonical Lys P2 site (Fig. 7C, D; Supplementary Fig. 4; see Supplementary Table 11 for data and refinement statistics, and Supplementary Table 14 for hydrogen bond interactions). The NLS site also shifted, with HKU5 binding at the major and minor sites with the same sequence, RKRRRHP^37^. The equivalent sequence in MERS-CoV ORF4b, RKRSHSP^28^, lacks two Arg residues. These results highlight that small changes within ORF4b of viruses within the subgenus *Merbecovirus* may result in significant changes in IMPα binding, indicating that a thorough investigation of the binding mechanisms of each would be needed to fully understand how they might influence immune evasion properties.

## Discussion

MERS-CoV ORF4b is a viral accessory protein that inhibits NF-κB-dependent innate immune responses in infected cells by binding preferentially to the nuclear import adapter IMPα3, one of seven isoforms in humans^11^. The structural data presented here showed that ORF4b binds to IMPαs using a novel interaction geometry and also provides insight into how MERS-CoV ORF4b inhibits innate immunity by impacting the nuclear import of NF-κB.

ORF4b has been reported to bind preferentially to the host nuclear import protein IMPα3, one of seven isoforms in humans^11^. Mechanisms through which cargoes are able to bind specifically to different IMPα isoforms are poorly understood and this question is complicated by the high degree of structural and sequence homology among IMPα isoform cargo binding regions^21^. To date, only three studies have provided a structural basis for specificity^21,27,28^. Here, by using a combination of structural, biophysical, and cellular data, we have examined the ORF4b interaction with different IMPα isoforms. Our structural data show an extensive interaction interface that involves residues within both of the previously identified sites 1 and 2 of the ORF4b NLS (docked at the minor and major NLS binding sites of IMPα, respectively), together with critical residues between these sites that had not previously been recognized as important (Fig. 1E, F). Paradoxically, this interaction interface was very similar in both the nuclear import adapter IMPα2 and in IMPα3, the latter of which ORF4b reportedly exhibited a binding preference^11^. Probing this interaction interface using in vitro binding studies showed a direct and high-affinity interaction of ~1–10 nM between the ORF4b NLS and representative members from all of the IMPα families (Fig. 1D), consistent with the extensive interface identified in the crystal structures. Moreover, examining these interactions within a cellular context, our immunoprecipitation experiments showed that IMPα isoforms from each subfamily were able to interact with MERS ORF4b (Figs. 1C and 4D–F), however these results do not necessarily dispute the hypothesis, nor previous studies, that full-length ORF4b may bind IMPα3 specifically. These same approaches have been used to determine mechanisms of isoform specificity for both viral proteins from our laboratory, including Hendra and Nipah virus W proteins^21^, and host transcription factors including SOX2 protein^27^, as well as studies from other laboratories including RCC1^28^. Additional specificity may be provided by other regions of ORF4b, or it may be that certain cell types or the context of an infected cell may alter ORF4b IMPα isotype specificity. Although further studies will be required to address this possibility, the structural and functional data presented here provide an important platform for such investigations.

Our structural data showed that the ORF4b NLS bound to IMPαs using a novel interaction geometry (Fig. 2). In the classical nuclear import pathway, cargo binding the major site of IMPα is highly dependent on a Lys at the P2 position that interacts with Gly^150^, Thr^155^, and Asp^192^ within the IMPα pocket. The terminal NZ nitrogen of the highly conserved P2 Lys is able to mediate an array of H-bonding interactions with all three residues that comprise the IMPα P2 binding pocket, including H-bonds with the main chain carbonyl group of Gly^150^ and the hydroxyl group of Thr^155^, as well as a salt bridge with the negatively charged Asp^192^ side chain. Numerous viral and host proteins have been shown to shuttle into the nucleus using this canonical P2 Lys site (Supplementary Fig 3). Surprisingly, MERS ORF4b does not contain a Lys at the P2 site, but rather a Val (position 35), which did not form any interactions with the Gly^150^, Thr^155^, and Asp^192^ pocket. Rather, MERS ORF4b bound IMPα2 and IMPα3 through an Arg residue (position 33, the equivalent of the P0 position) at this site and formed similar interactions to those made by a Lys NZ nitrogen at the P2 site. Because of this binding mechanism was so unusual, we examined all IMPα structures deposited to the Protein Data Bank, where we found that only one other cargo bound to IMPα using a P0 Arg. The Epstein‐Barr virus nuclear antigen leader protein (EBNA‐LP) was shown to bind using a highly similar interaction, in which a P0 Arg binds at the canonical P2 site (Supplementary Fig. 3). Interestingly, both proteins contain a Val at the P2 site, and future studies are required to examine whether an RxV motif is important for this binding mechanism, and the extent to which other viruses or host nuclear cargo utilize this binding motif. One notable difference between MERS-CoV ORF4b and EBNA‐LP is that the latter bound only at the major site, contrasting with MERS-CoV ORF4b that bound at both the major and minor sites of IMPα. Together these data suggest that the RxV motif can mediate mono- and bi-partite NLS interactions.

Intriguingly, mutations with the NLS of MERS-CoV ORF4b resulted in a dramatic shift towards canonical Lys P2 binding (Fig. 3). For example, mutations within both the minor site (Arg24Ala), and the major site (Arg33Ala and Arg37Ala) resulted in the MERS-CoV ORF4b binding using a canonical Lys P2 involving ^29^TKKLR^33^, with Lys^30^ binding at the P2 site. These NLSs all bound with reduced affinity compared to the Arg P0 site. Consistently, although mutations still allowed interaction with IMPα at the interface using the Lys P2, both the level of nuclear import and the ability to interfere with innate immunity were reduced (Figs. 5 and 6E, F and Supplementary Figs. 10 and 11). This illustrates how small mutations within viruses can result in different binding geometries to IMPα with associated functional consequences, and that sequence alignment alone cannot be used with great confidence to predict IMPα binding interfaces.

Our data also provides a structural basis for understanding how the MERS-CoV ORF4b inhibits innate immunity by impacting the nuclear import of NF-κB. Both the p50 and p65 components of the NF-κB heterodimer contain NLSs and it has been proposed that the p50 NLS binds to the major NLS-binding site on IMPαs whereas the p65 NLS binds to the minor site^26^. Our crystal structures confirm that the binding site for the NLS region of p50 is located within the major site of IMPα, overlapping directly with the high-affinity P0 Arg binding site of ORF4b (Fig. 6A–D). We confirm that ORF4b reduces innate immune responses including NF-κB and IFNβ induction, and that mutations within the ORF4b interface reduce this activity. This finding supports the published work of Canton et al. (2018) who observed an inhibitory mechanism dependent upon direct competition between MERS-CoV-expressed ORF4b and endogenous NF-κB subunits for binding to IMPα and subsequent nuclear import. Whether ORF4b inhibition of IRF3 and IRF7-mediated IFNβ induction (as suggested within the literature^7,14^) occurs via an analogous mechanism remains to be explored.

Future studies will be aimed towards understanding how ORF4b proteins of related merbcoviruses bind to IMPα. In this regard, we have examined the binding mechanism of the HKU5 ORF4b NLS bound to IMPα, which indeed uses a canonical Lys P2 binding mechanism (Fig. 7C, D). Our data confirms the results from an earlier study which demonstrated that downstream “site 2” basic residues are unimportant for HKU5 ORF4b nuclear localization^7^. Indeed, despite the complete conservation of the downstream KRR motif among merbecovirus ORF4b proteins (Fig. 7B and Supplementary Fig. 12), these are not utilized for HKU5 ORF4b interactions with IMPα2; rather the functional NLS comprises the mono-partite sequence ^31^RKRRR^35^ which was capable of binding at both the major and minor sites on IMPα. Intriguingly, this monopartite “site 1”-only NLS has been shown to still be sufficient for the inhibition of NF-κB-mediated innate immune signaling^7^.

An earlier study suggested that an altered ORF4b:IMPα interaction modulates the efficiency of PDE domain-mediated inhibition of the OAS/RNase L pathway^10^. When each were expressed recombinantly from an NS2-inactive mouse hepatitis virus (MHV) vector, HKU5 ORF4b localized to the nucleus to a greater extent than MERS-CoV ORF4b^10^ (for which a small proportion of protein remained cytoplasmic, as has been observed in the current study (Fig. 5)). However, the authors reported that RNase L-dependent RNA cleavage was still observed in MHV-HKU5 ORF4b infected cells, contrasting with inhibition of this pathway seen when WT and NLS-deleted (Δ1-52) MERS-CoV ORF4b were expressed^10^. This suggests that the activity of the ORF4b NLS region may have to strike a balance between nuclear import to competitively inhibit host immune transcription factors, and retention within the cytoplasm to allow PDE domain inhibition of OAS/RNase L. Further studies are required to determine whether the non-canonical Arg P0 IMPα-binding mechanism somehow mediates this balanced innate immune antagonism phenotype.

Finally, it should be noted that both SARS-CoV-1 and 2 (genus *Betacoronavirus*, subgenus *Sarbecovirus*), although related to MERS-CoV (genus *Betacoronavirus*, subgenus *Merbecovirus*), lack the ORF4b gene. Instead, SARS-CoV-1 and 2 inhibit nuclear import of innate immune signaling proteins using ORF6. Although ORF4b and ORF6 are unrelated, both are tasked with the function of preventing innate immune signals from reaching the nucleus. In the case of SARS-CoV-1, ORF6 was demonstrated to antagonize STAT1 functioning by tethering IMPα1 to the rough endoplasmic reticulum/Golgi membrane^29^. This activity mapped to the C-terminal region of ORF6 and bears no similarity to ORF4b. In SARS-CoV-2, it was demonstrated ORF6 localized to the nuclear pore complex, and disrupted cargo-receptor docking^30^. Together this suggests that although betacoronaviruses have evolved different mediators of innate immune pathway inhibition, mechanisms that suppress IMPα transport of transcription factor cargo are a common virulence determinant among pathogenic viruses within this group.

## Methods

### Protein expression and purification for structural and binding studies

A gene fragment encoding the MERS-CoV ORF4b NLS region (NC_019843.3 residues 19–39) and an N-terminal TEV cleavage site was codon optimized for bacterial expression and synthesized by Genscript and cloned into the pGEX4T vector at the BamHI site. The NF-κB p50 NLS region (UniProtKB P19838; residues 331–370) was codon optimized for expression in E. coli, synthesized with an added N-terminal TEV cleavage site, and cloned into pGEX4T-1 vector at BamHI and EcoRI sites (Genscript, Piscataway, NJ). Constructs encoding IMPαs lacking the IBB domain and an N-terminal TEV cleavage site, were codon optimized and synthesized by Genscript, and cloned into pET30a using the BamHI site, with the following accession numbers: IMPα1 (UniProtKB P52292; residues 70-529); IMPα2 (UniProtKB P52293; residues 70-529), IMPα3 (UniProtKB 000629; residues 64–521), IMPα5 (UniProtKB p52294, residues 73–538), and IMPα7 (UniProtKB encoding residues 73–536). Plasmids were transformed into BL21 (DE3) pLysS cells and expressed as described previously^31^. Purification of 6xHis tagged IMPα proteins were performed by injecting clarified cell lysate onto a HisTrap 5 mL column using binding buffer (50 mM phosphate buffer pH 8.0, 300 mM NaCl, 20 mM imidazole). The column was washed with 15 column volumes of binding buffer then eluted using a gradient elution over 5 column volumes to reach 100% elution buffer (50 mM phosphate pH 8.0, 300 mM NaCl, 500 mM imidazole). Samples were pooled, and the affinity tag removed by TEV proteolysis. Size exclusion purification of pooled samples was performed on a Superdex 200 pg 26/600 column using gel filtration buffer (50 mM Tris, 125 mM sodium chloride, pH 8.0). Eluted proteins were pooled and concentrated to 10–20 mg/ml using 10 kDa MW centrifuge filters.

Complexes of MERS-CoV ORF4b NLS and IMPα isoforms were achieved by mixing lysed E. coli cells expressing GST-tagged MERS-CoV ORF4b NLS with E. coli cells expressing IMPα isoforms. Purification of complexed proteins were performed by passing cleared lysate over a HisTrap 5 mL column using binding buffer (50 mM phosphate buffer pH 8.0, 300 mM NaCl, 20 mM imidazole), washing with 15 column volumes of binding buffer, and elution using a gradient elution over 5 column volumes to reach 100% elution buffer (50 mM phosphate pH 8.0, 300 mM NaCl, 500 mM imidazole). Samples were pooled, and the His and GST affinity tag removed by TEV proteolysis. Size exclusion purification of pooled samples was performed on a Superdex 200 pg 26/600 column using gel filtration buffer (50 mM Tris, 125 mM sodium chloride, pH 8.0). The protein was then passed through a 5 ml GSTrap FF column to remove GST, and the unbound protein complex was concentrated to 10–20 mg/ml using 10 kDa MW centrifuge filters, aliquoted, and flash-frozen in liquid nitrogen and stored at −80 °C.

### Crystallization, data collection, and processing

All crystals were obtained using the hanging drop vapor diffusion method over a 300 μL reservoir solution. IMPα2: MERS-CoV NLS was crystallized in 700 mM sodium citrate (pH 7.0) and 10 mM DTT. Single rod-shaped crystals forming within 3 days. IMPα3: MERS-CoV NLS crystallized in 0.2 M magnesium chloride, 0.1 M Tris pH 8.0, 20% w/v PEG 6000 with plate-shaped crystals forming within 14 days. Crystallization of IMPα2 in the presence of MERS ORF4b NLS wild-type and mutant peptides was achieved by mixing the peptide in a 2:1 molar ratio, and crystallization conditions were the same as the protein complex above. IMPα2 bound to NF-κB p50 was crystallized in 700 mM sodium citrate, 10 mM DTT, 100 mM sodium HEPES pH 7.0, with rod-shaped crystals appearing in 2–3 days. IMPα3 bound to NF-κB p50 was crystallized in 0.2 M potassium thiocyanate and 20% w/v PEG 3350, with rod-shaped crystals appearing in 2–3 days. X-ray diffraction data were collected at the Australian Synchrotron on the MX1 and MX2 macromolecular beam lines using Eiger2 9 M and Eiger 16 M detectors, respectively^32,33^. Data reduction and integration was performed using iMosflm^34^ and XDS^35^. Merging, space group assignment, scaling and selection of 5% reflections for Rfree calculations were performed using Aimless^36^ within the CCP4 suite^37^. Phasing was performed using molecular replacement in Phaser MR, with PDB codes 7JJM and 6BVZ used as a search model for IMPα2 and IMPα3 respectively^21,38^. Models were built and refined using iterative cycles of coot^39^ and maximum likelihood phenix refine^40^. The final models have been validated and deposited to the PDB with accession numbers detailed in Supplementary Tables.

### Electrophoretic mobility shift assays

FITC labeled MERS-CoV ORF4b NLS peptides (8.5 μl of 0.5 mg/ml) were mixed with 40 μM of each IMPα isoform and incubated in a total volume 17 μl for 15 min at room temperature. Samples were supplemented with 3 μl of 50% glycerol and run on a 1% agarose TB gel for 1.5 h at 70 V in TB running buffer. The images were recorded using a SYBR green filter within a Gel Doc BioRad Gel Doc imaging system. The gel was then stained using Coomassie stain and destained in 10% ethanol and 10% glacial acetic acid.

### Fluorescence polarization assays

FITC-tagged MERS ORF4b peptides (5 nM) were incubated with two-fold serially diluted IMPα concentrations (starting concentration 5 µM) across 23 wells to a total volume of 200 µL. Fluorescence polarization measurements were recorded using a CLARIOstar Plus plate reader (BMG Labtech) in 96-well black Fluotrac microplates (Greiner Bio-One; Kremsmünster, Austria). Assays were repeated in triplicate and contained a negative control (no binding partner) and blank (Tris buffered saline, pH 8). Triplicate data was fitted to a single binding curve using GraphPad Prism.

### Cell culture and plasmids

HEK293T (ATCC—CRL-3216) and Huh7 cells (a generous gift from the Gordan lab at University of California at San Francisco) were maintained in Dulbecco’s Modified Eagle Medium, supplemented with 10% fetal bovine serum and cultured at 37 °C and 5% CO_2_.

The codon-optimized sequence for MERS ORF4b (NC_019843.3), was synthesized (Genscript, Piscataway, NJ) and cloned with an N-terminal Flag- or HA-tag into the mammalian expression plasmid pCAGGS. Flag-tagged MERS ORF4b mutants R24A, R33A, R24A/R33A and RKRA (R22A/K23A/R24A) were generated by overlapping PCR. MERS ORF4b delta38 was cloned using PCR to remove the first 38 amino acids. pCAGGS Flag- and HA-tagged IMPα1, 3, 4, 5, 6, and 7 have previously been described^16^.

### Immunofluorescence assays

MERS ORF4b nuclear translocation: Huh7 cells (3 × 10^4^) grown on glass coverslips were transfected with indicated wt and mutant Flag-tagged MERS ORF4b plasmids (500 ng) using Lipofectamine 2000 (Thermo Fisher Scientific, MA). At 24 h post transfection, cells were fixed using 4% paraformaldehyde and permeabilized using 0.1% Triton X-100. Cells were stained using anti-Flag (DYKDDDK) tag monoclonal antibody conjugated to Alexa Fluor 488 (Invitrogen, cat# MA1-142-A488, lot# UB276601) (dilution 1:1000). To determine the ratio of nuclear to cytoplasmic fluorescence signal (Fn/c), coverslips were imaged using a BioTek Cytation 5 Cell Imaging Multi-Mode reader. Images were analyzed using Gen5 Image Prime software to determine Fn/c, using the calculation Fn/c = (Fn-background)/(Fc-background), where Fn is nuclear fluorescence and Fc is cytoplasmic fluorescence. Fn/c was determined for ≥60 cells in each condition; error bars indicate the mean values ± standard deviation. Images of the same coverslips were taken using a Zeiss LSM 800 confocal microscope at ×64.

p65 nuclear translocation: Huh7 cells were transfected as above with the indicated Flag-tagged MERS ORF4b plasmids or empty vector. At 24 h post-transfection, media was changed to DMEM without FBS for four hours, after which coverslips were treated with 20 ng/ml of TNFα (Peprotech) for 30 min. Coverslips were fixed and permeabilized as above and cells were stained with anti-p65 (Santa Cruz, cat# sc-109, lot# F2304) (dilution 1:100) and goat anti-rabbit IgG Alexa Fluor 647 (Invitrogen, cat# A-21244, lot# 1871168) (dilution 1:1000), and anti-Flag Alexa Fluor 488 (Invitrogen, cat# MA1-142-A488, lot# UB276601) (dilution 1:1000). Fn/c for p65 was determined as above using a BioTek Cytation 5 Cell Imagining Mult-Mode reader and Gen5 Image Prime software, selecting for cells expressing MERS ORF4b or mutants in transfected cells. Fn/c was determined across five fields of view for ≥24 cells expressing MERS ORF4b or mutants, and across three fields of view for ≥139 cells for −TNFα and +TNFα controls.

### Co-immunoprecipitation assay

HEK293T cells (1 × 10^6^) were transfected with the indicated plasmids using Lipofectamine 2000 (Thermo Fisher Scientific). At 24 h post transfection, cells were lysed in NP-40 lysis buffer (50 mM Tris pH 7.5, 280 mM NaCl, 0.5% NP-40, 0.2 mM EDTA, 2 mM EGTA, 10% glycerol, protease inhibitor (complete; Roche, Indianapolis, IN)). Anti-FLAG M2 magnetic beads or EZview Red anti-HA agarose affinity gel (Sigma-Aldrich) were incubated as indicated with lysates for 1 h at 4 °C, washed five times in NP-40 lysis buffer, and eluted using 3X FLAG or HA peptide (Sigma-Aldrich) at 4 °C for 30 min or by boiling in sample buffer for five minutes. Whole cell lysates (4% of sample) and co-precipitation samples (25% of sample) were analyzed by western blot.

### Western blots

Lysates were run on 10% Bis-Tris Plus polyacrylamide gels (Thermo Fisher) and transferred to PVDF membrane (Bio-Rad). Membranes were probed with the indicated antibodies and were developed by Western Lightning Plus ECL (Perkin Elmer) and imaged on a ChemiDoc MP Imaging System (Bio-Rad). Antibodies, rabbit anti-Flag (cat# F7425), and mouse anti-β-tubulin (cat# T8328) were purchased from Sigma-Aldrich. Rabbit anti-HA (cat# 71-5500) and mouse anti-GAPDH (GA1R) (cat# MA5-15738) were purchased from Invitrogen. Anti-rabbit and anti-mouse IgG, HRP-linked antibodies were purchased from Cell Signaling (cat# 7074 and 7076).

### Reporter assays in HEK293T cells

For the Sendai virus (SeV)-induced IFNβ promoter luciferase assay, HEK293T cells (7.5 × 10^4^ cells/well) were transfected with 30 ng of the IFNβ firefly luciferase reporter, 30 ng of a constitutively expressed Renilla luciferase reporter (pRLTK), and Flag-tagged MERS ORF4b and mutants (25 ng and 2.5 ng), using Lipofectamine 2000 (Thermo Fisher Scientific). Twenty-four hours post transfection, cells were infected with 200 HA units (HAU) of SeV for 18 h. Luciferase activity was assessed using a dual-luciferase assay (Promega) and read on an EnVision multilabel plate reader (PerkinElmer, Inc.). Firefly luciferase values were normalized to Renilla luciferase values. The assay was performed in triplicate; error bars indicated the mean values ± standard deviation for the triplicate. For the NF-κB promoter luciferase assay, HEK293T cells (7.5 × 10^4^ cells/well) were transfected with 30 ng of the NF-κB firefly luciferase reporter, 30 ng of a constitutively expressed Renilla luciferase reporter (pRLTK), and Flag-tagged MERS ORF4b and mutants (25 ng and 2.5 ng), using Lipofectamine 2000 (Thermo Fisher Scientific). Twenty-four hours post transfection, cells were treated with 10 ng/ml of TNFα (PeproTech, cat# 300-01A) for six hours. Luciferase activity was assessed and analyzed as described above.

### Reporter Assays in A549 cells

For the IFNβ reporter assay, A549 cells (4 × 10^4^ cells/well) were transfected with 30 ng of the IFNβ firefly luciferase reporter and 30 ng of a constitutively expressed Renilla luciferase reporter plasmid (pRLTK). Additionally, 25 ng or 2.5 ng of Flag-tagged MERS WT ORF4b (ORF4b), ORF4b R24A (R24A), ORF4b R33A (R33A), or ORF4b R24A/R33A (R24A/R33A) were transfected using TransIT-LT1 (Mirus). Twenty-four hours post-transfection, cells were infected with 200 hemaglutinating units (HAU) of SeV for 18 h. Luciferase activity was assessed using a dual-luciferase assay (Promega) and read on an EnVision multilabel plate reader (PerkinElmer, Inc.). Firefly luciferase values were normalized to Renilla luciferase values. The assay was performed in triplicate; error bars indicate the mean values ± standard deviation for the triplicate. For the NFκB reporter assay, the transfection was performed as above, using 30 ng of the NFκB firefly luciferase reporter. Twenty-four hours post-transfection, cells were treated with 50 ng/ml of TNFα (PeproTech, cat# 300-01 A) for 18 h. Luciferase activity was assessed and analyzed as described above.

### Phylogenetic analyses

Phylogenetic analysis of betacoronavirus sequences was performed using Geneious Prime Java Version 11.0.4 + 11 (64 bit). Complete genome nucleotide sequences of 10 viruses (GenBank accession numbers HM211099.1, NC_039207.1, KC869678.4, MK907287.1, KX442565.1, NC_009019.1, NC_009020.1, NC_019843.3, MG923475.1, and KJ650295.1) were aligned using MAFFT v7.450^41^, with the resulting alignment subsequently used to build a maximum likelihood PhyML (v3.3.20180621) tree using the HKY85 substitution model and bootstrapping of 1,000 replicates^42^. The resulting tree was rooted on the Bat norbecovirus (HM211099.1) outgroup. Full-length amino acid sequences of the ORF4b protein from 9 merbecoviruses corresponding to the full-length genomes (GenBank accession numbers AHX00714.1, AIG13099.1, ASL68956.1, AVN89394.1, QGA70705.1, YP_001039956.1, YP_001039965.1, YP_009047207.1, and YP_009513014.1) were also aligned using MAFFT v7.450. The aligned amino acids 59–81 were subsequently used to generate a sequence logo using WebLogo 3^43^, and colored based upon amino acid charge.

### Statistical analyses

Statistical analysis was performed using GraphPad Prism 9^44^. Data points were considered significantly different if the *P* value was <0.05. Further details can be found in the figure legends.

### Reporting summary

Further information on research design is available in the Nature Research Reporting Summary linked to this article.

## Supplementary information

Supplementary Information

Reporting Summary

## Data Availability

Protein Data Bank files associated with the structures generated in this study have been deposited to the Protein Data Bank and were released prior to review of the manuscript. The codes are IMPα2:MERS ORF4b 7RFX; IMPα3:MERS ORF4b 7RFY; IMPα2:MERS ORF4b wt peptide 7RFZ; IMPα2:MERS ORF4b R24A peptide 7RG0; IMPα2:MERS ORF4b H26A peptide 7RG1; IMPα2:MERS ORF4b R33A peptide 7RG2; IMPα2:MERS ORF4b R37A peptide 7RG3; IMPα2:NF-κB p50 7RG4; IMPα3:NF-κB p50 7RG5; and IMPα2:HKU5 ORF4b 7RG6. Source data are provided with this paper.

## Source data

Source Data
