# Supplementary material for: MERS-CoV ORF4b employs an unusual binding mechanism to target IMPα and block innate immunity
Source: Nat Commun. 2022 Mar 25;13:1604. doi: 10.1038/s41467-022-28851-2 (PMC8956657; doi:10.1038/s41467-022-28851-2)
Supplement: Supplementary file 1 — Supplementary Information [file 41467_2022_28851_MOESM1_ESM.pdf]

Supplementary information for STRUCTURAL CHARACTERIZATION OF MERS-CoV ORF4b BINDING TO IMPα REVEALS AN UNUSUAL BINDING MECHANISM

Fig 1C Uncut blot

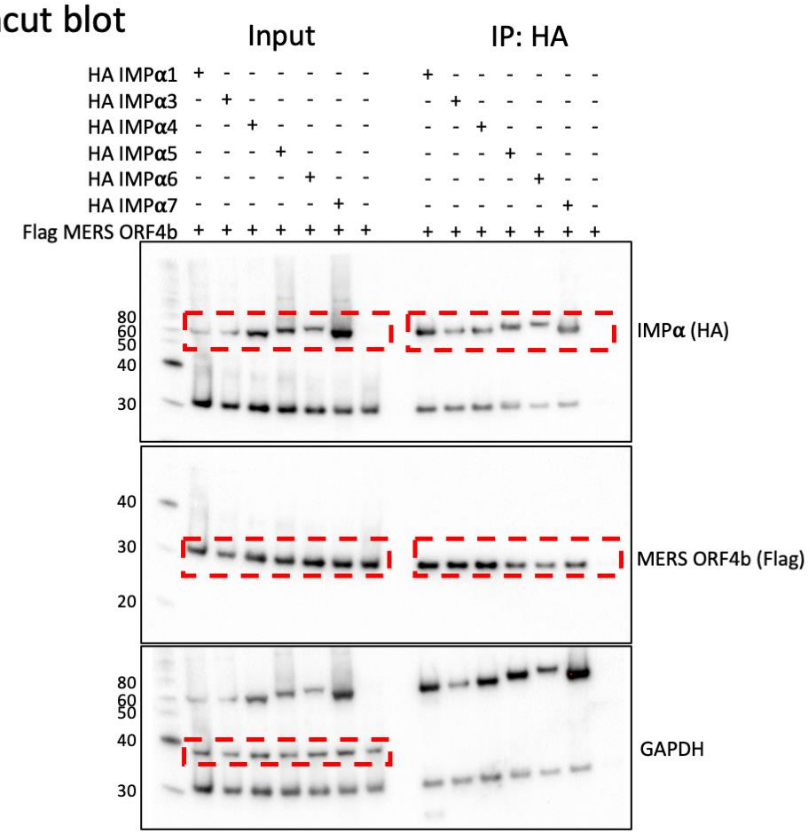

Supplementary Fig 1. Uncut Western Blots shown in Fig 1C of the manuscript.

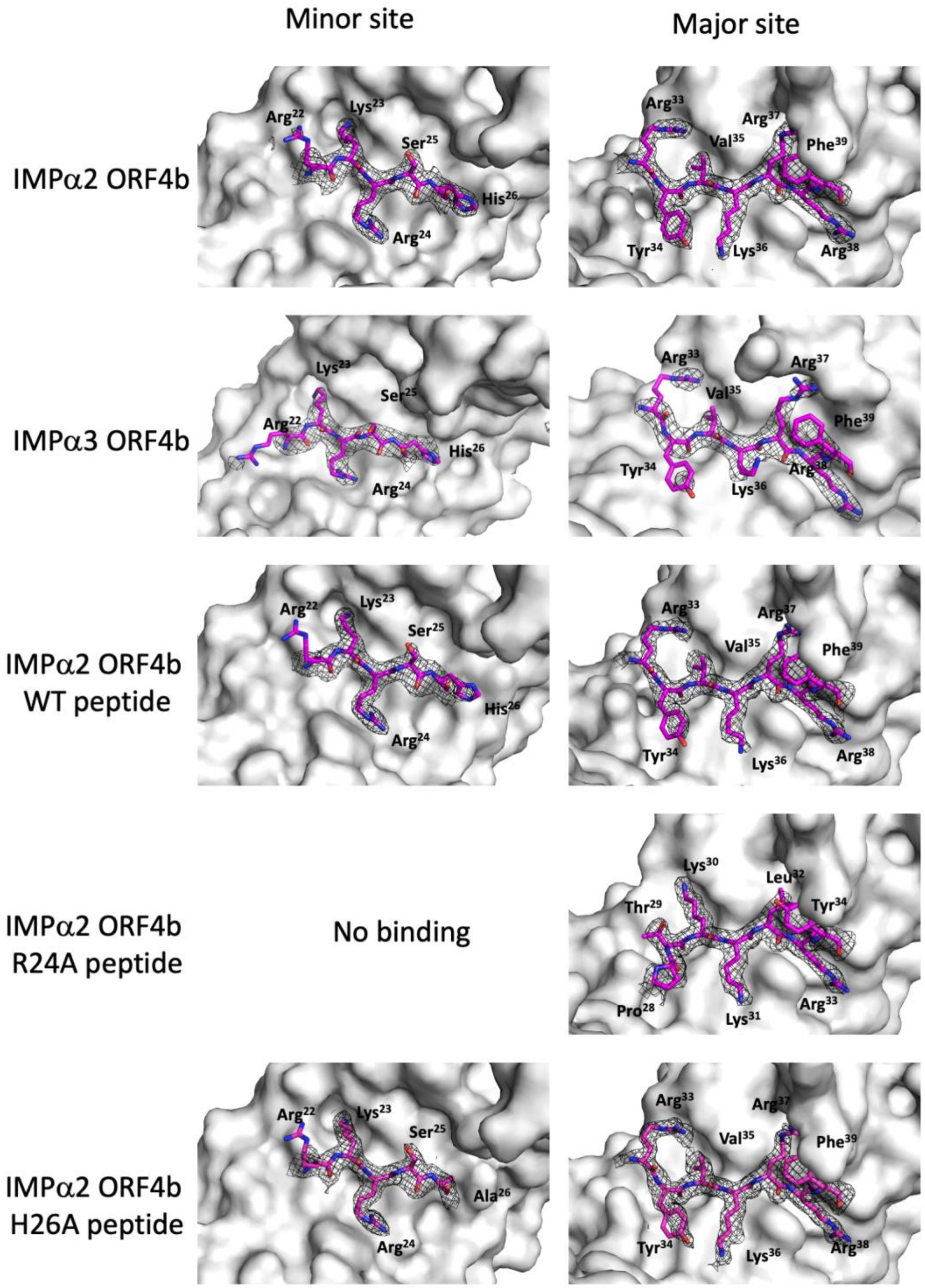

Supplementary Fig 2. Crystal structures of IMPα isoforms bound to MERS-CoV ORF4b NLS regions including mutants. IMPα is shown in surface view and colored light grey, and the cargo in stick mode, colored purple. Pymol software was used to generate images[1]. Associated 2Fo-Fc maps of the cargo are contoured to 1σ in black.

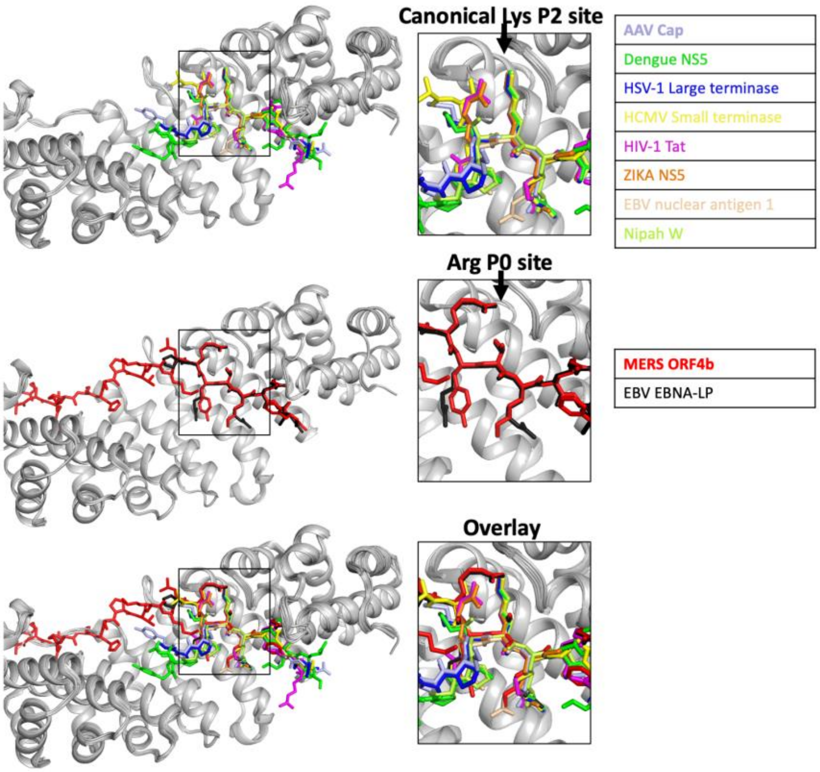

PDB	Viral protein	P0	P1	P2	P3	P4	P5
7L04	AAV Cap	K	K	K	K	A	R
5FC8	Dengue NS5	S	M	K	R	F	R
5HUW	HSV-1 Large terminase	P	K	K	R	A	K
5HUY	HCMV Small terminase	T	R	K	P	R	R
5SVZ	HIV-1 Tat	G	R	K	K	R	R
5W41	ZIKA NS5	-	R	K	R	P	R
5WUN	EBV nuclear antigen 1	-	E	K	R	P	R
6BW0	Nipah W	P	T	K	K	A	R
MERS	MERS ORF4b	R	Y	V	K	R	R
5X8N	EBV EBNA-LP	R	R	V	R	R	R

Supplementary Fig 3. Crystal structures of IMPα isoforms bound to viral cargo. A Lys P2 site is a signature binding motif of NLS regions (top panel). These include Adeno-associated virus Capsid protein[2], Dengue NS5 protein[3], Herpes simplex virus large terminase[4], Human Cytomegalovirus large terminase[4], Human immunodeficiency virus Tag[5], ZIKA virus NS5[6], Epstein-Barr virus nuclear antigen 1[7], Nipah virus W[8]. An unusual binding mechanism was identified in MERS ORF4b (middle panel), where a P2 Lys is replaced by a Val. An Arg at P0 site mediated equivalent binding. A similar mechanism was observed in Epstein-Barr virus nuclear antigen leader protein[9]. An overlay is presented in the bottom panel.

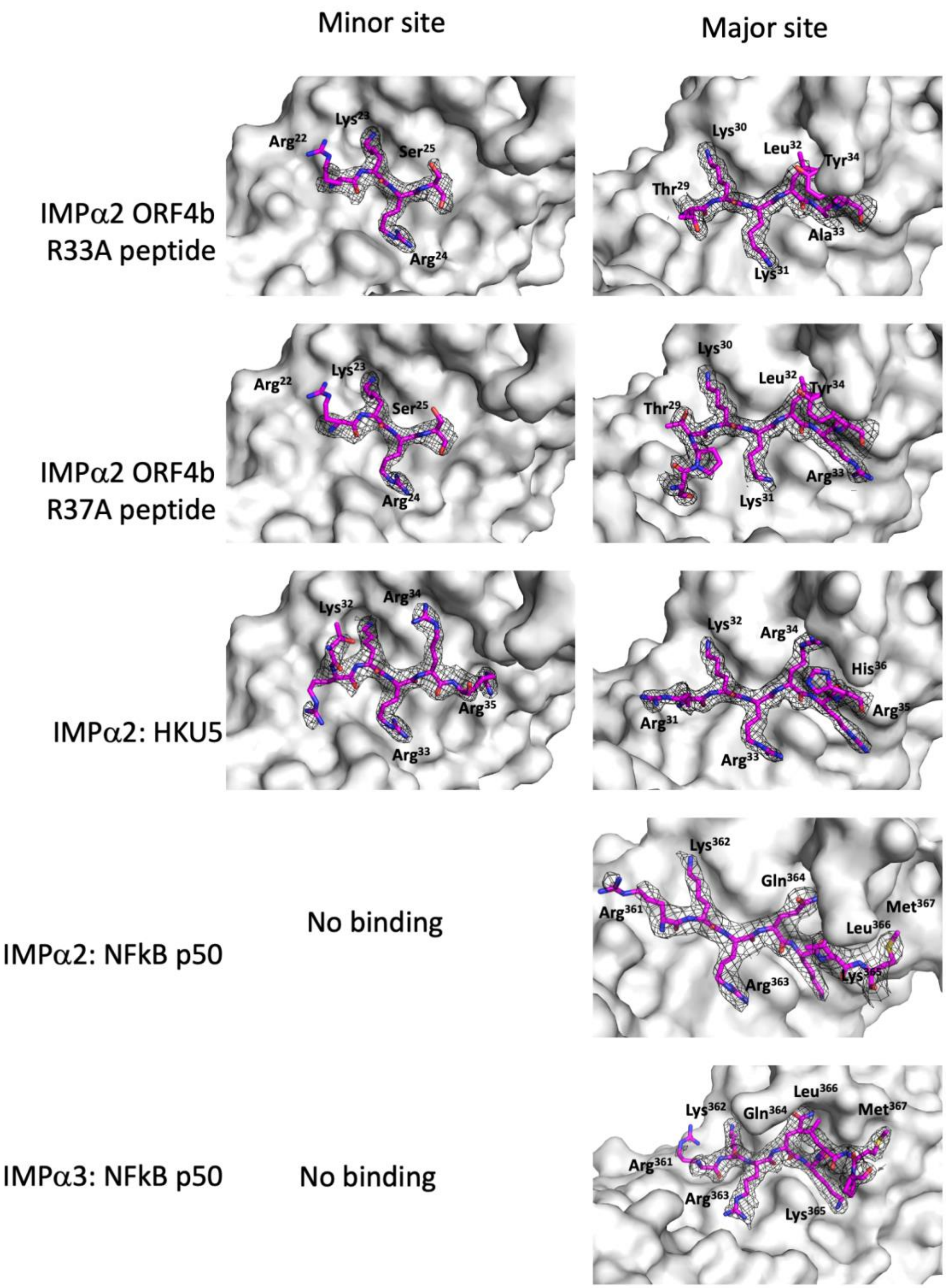

Supplementary Fig 4. Crystal structures of IMPα isoforms bound to MERS-CoV ORF4b NLS regions including mutants, and NF-κB p50. IMPα is shown in surface view and colored light grey, and the cargo in stick mode, colored purple. Pymol software was used to generate images[1]. Associated 2Fo-Fc maps of the cargo are contoured to 1σ in black.

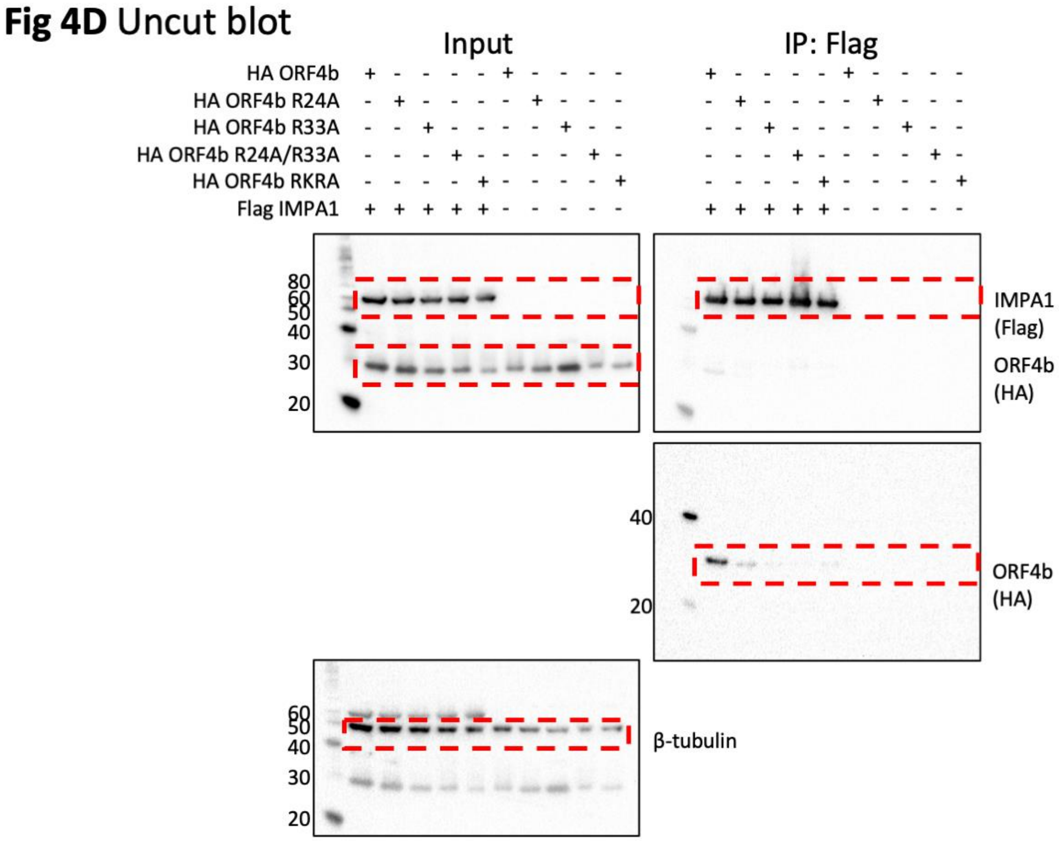

Supplementary Fig 5. Uncut Western Blots shown in Fig 4D of the manuscript.

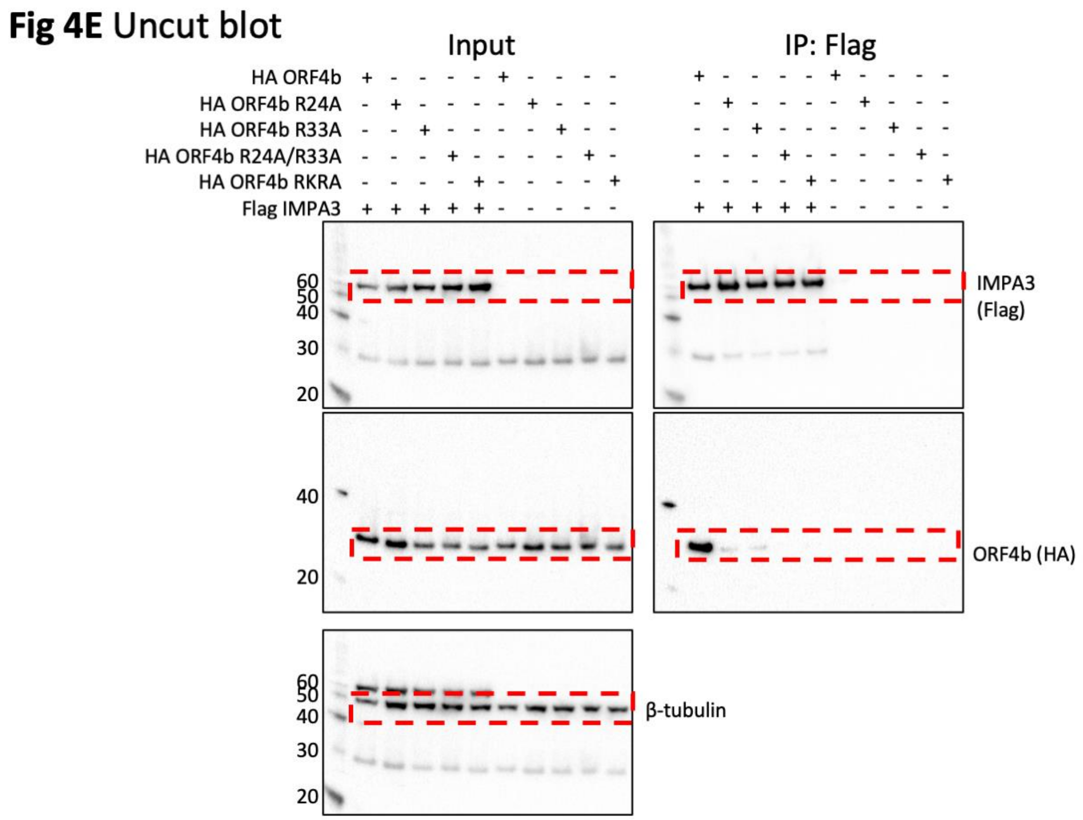

Supplementary Fig 6. Uncut Western Blots shown in Fig 4E of the manuscript.

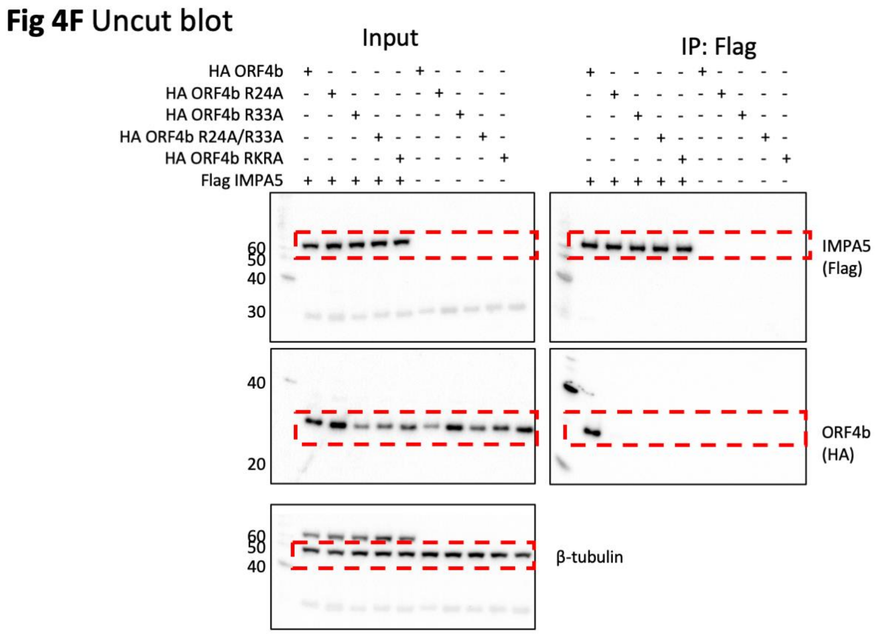

Supplementary Fig 7. Uncut Western Blots shown in Fig 4F of the manuscript.

Fig 6E Uncut blot

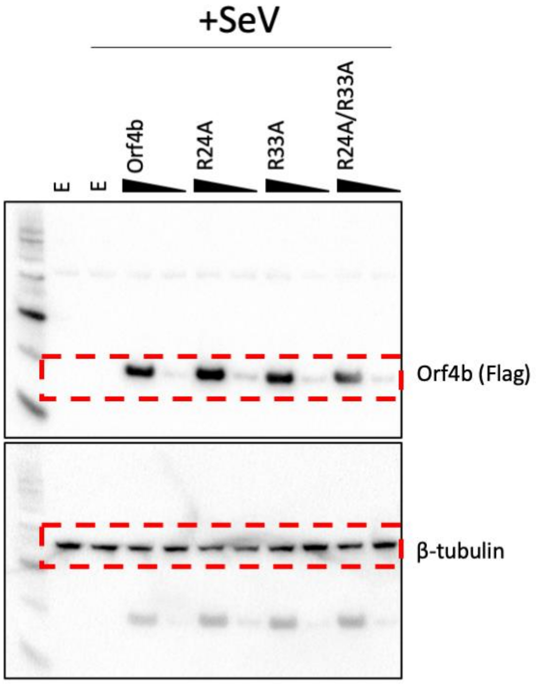

Supplementary Fig 8. Uncut Western Blots shown in Fig 6E of the manuscript.

Fig 6F Uncut blot

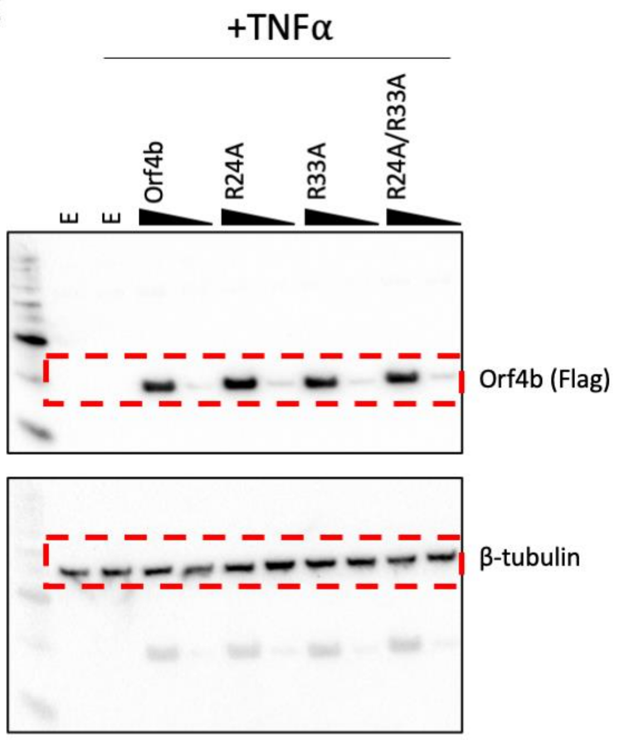

Supplementary Fig 9. Uncut Western Blots shown in Fig 6F of the manuscript.

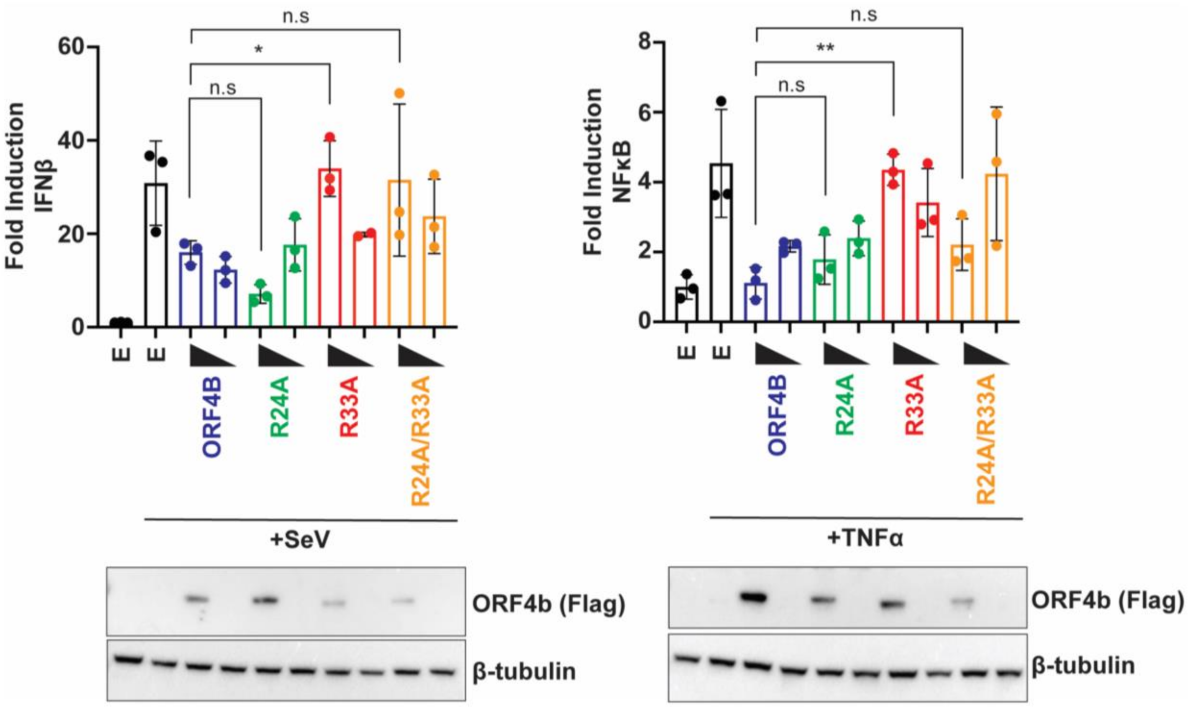

Supplementary Figure 10. ORF4b Suppresses IFNβ and NFκB Reporter Activity in the lung epithelial cell line A549. A) IFNβ reporter assay in A549 cells to test the inhibition of the IFNβ promoter by MERS WT ORF4b (ORF4b), ORF4b R24A (R24A), ORF4b R33A (R33A), or ORF4b R24A/R33A (R24A/R33A) 18 hours post-infection with Sendai virus (SeV). Empty vector (E), mock-infected and SeV-infected, served as controls. Values are reported as fold-induction relative to the Empty vector, mock-infected control. The error bars represent the standard deviation for triplicate experiments. n=3 biologically independent experiments. Statistical significance was determined by a one-way ANOVA followed by Tukey's test; n.s., no significance; *, p=0.0423. B) NFκB reporter assay in A549 cells to test the inhibition of the NFκB promoter by MERS WT ORF4b (ORF4b), ORF4b R24A (R24A), ORF4b R33A (R33A), and ORF4b R24A/R33A (R24A/R33A) following treatment with TNFα. Empty vector (E), mock-infected and SeV-infected, served as a controls. Values are reported as fold-induction relative to the Empty vector, mock-infected control. The error bars represent the mean values +/- standard deviation for triplicate experiments. n=3 biologically independent experiments. Statistical significance was determined by a one-way ANOVA followed by Tukey's test; n.s., no significance; **, p=0.003.

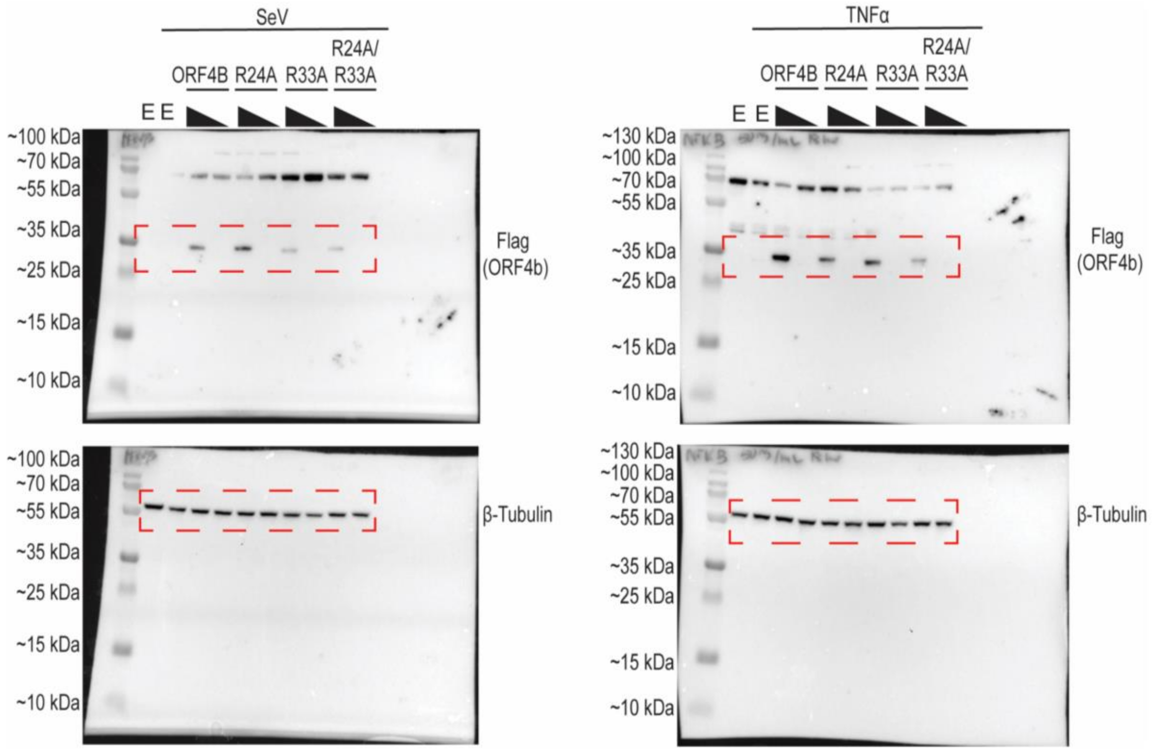

Supplementary Fig 11. Uncut Western Blots shown in Supplementary Figure 10 of the manuscript.

Supplementary Fig 12. Confocal microscopy images captured using a Zeiss LSM 800 confocal microscope of Huh7 cells transfected with Flag-tagged MERS-CoV ORF4b plasmids or empty vector. Cells were stained with rabbit anti-p65 and goat anti-rabbit IgG Alexa Fluor 647 (red), anti-DYKDDDK Alexa Fluor 488 (green), and DAPI (blue). The experiment was performed independently twice, with similar results. Scale bar is 10 μm.

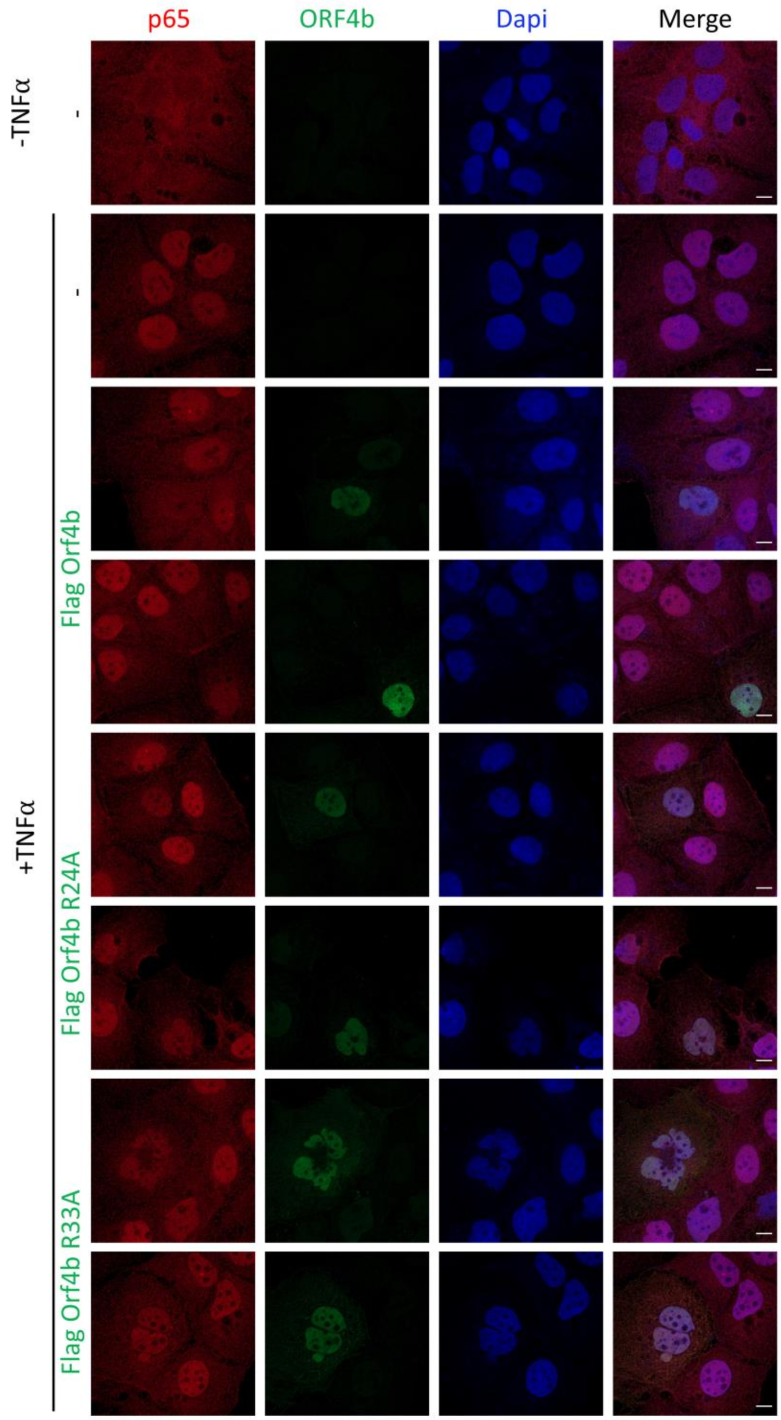

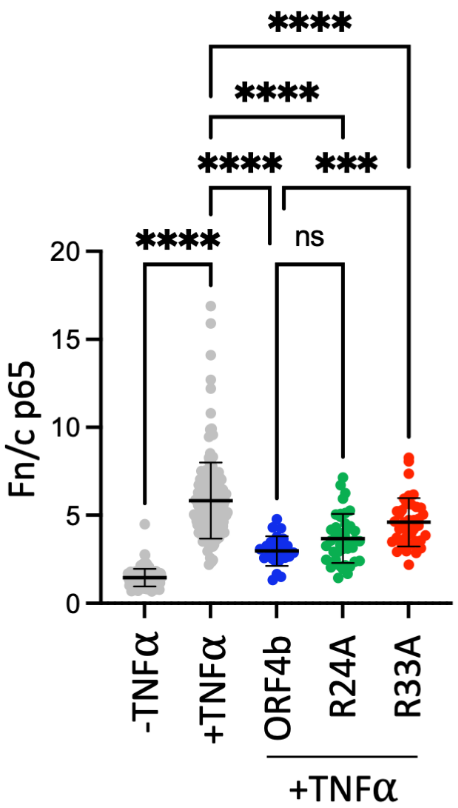

Supplementary Fig 13. Quantification of the nuclear to cytoplasmic fluorescence signal (Fn/c) of NF-κB p65 (Supplementary Fig 8) was determined across five fields of view for cells expressing MERS-CoV ORF4b (n = 24 cells), ORF4b R24A (n = 40 cells), ORF4b R33A (n = 39 cells), and across three fields of view for -TNFα (n = 139 cells) and +TNFα (n = 169 cells) controls, with error bars indicating the mean values +/- standard deviation. NS = no significant difference, **** = $p < 0.0001$, *** = $p < 0.001$.

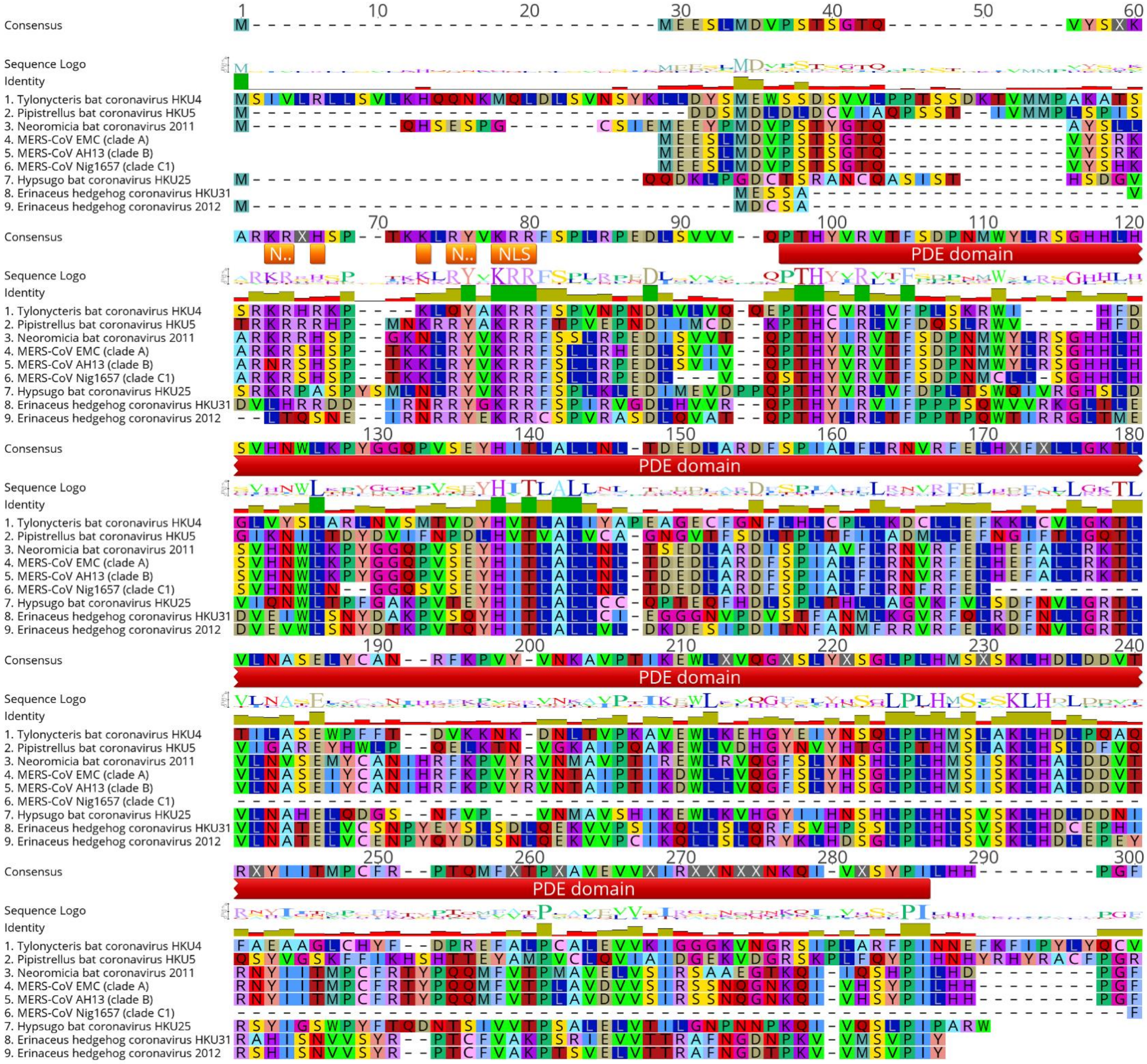

Supplementary Figure 14. MAFFT multiple sequence alignment of selected merbecovirus ORF4b amino acid sequences (GenBank accession numbers AHX00714.1, AIG13099.1, ASL68956.1, AVN89394.1, QGA70705.1, YP_001039956.1, YP_001039965.1, YP_009047207.1, and YP_009513014.1) generated in Geneious Prime. The position of residues determined in this study as participating in MERS-CoV ORF4b NLS interactions with IMPA proteins are annotated in orange, and the PDE domain is annotated in red. A consensus sequence is displayed, as well as a sequence logo and a graphical representation of identity.

Supplementary Table 1. Data collection and refinement statistics.

Data collection and processing	ORF4b:IMPα2	ORF4b:IMPα3
Wavelength (Å)	0.9537	0.9537
Resolution range (Å)	26.93 - 2.10 (2.16 - 2.10)	25.99 - 2.50 (2.60 - 2.50)
Space group	P 21 21 21	P 1 21 1
Unit cell (Å, ^{o})	79.31 89.68 99.36 90 90 90	48.22 59.71 82.64 90 98 90
Total reflections	207746 (17540)	65232 (7167)
Unique reflections	42012 (3409)	15900 (1762)
Multiplicity	4.9 (5.1)	4.1 (4.1)
Completeness (%)	99.8 (99.8)	98.2 (97.7)
Mean I/sigma(I)	4.9 (1.4)	5.4 (1.2)
Wilson B-factor Å^2	35.87	56.57
R-merge	0.16 (0.82)	0.11 (0.87)
R-pim	0.11 (0.57)	0.089 (0.72)
Refinement		
Number of reflections	41935	15841 (1539)
Number of R-free reflections	2019	785 (71)
R-work %	19.7	24.9
R-free %	20.8	26.8
RMS(bonds)	0.002	0.003
RMS(angles)	0.54	0.60
Ramachandran plot		
favored (%)	98.64	97.22
allowed (%)	1.36	2.78
outliers (%)	0	0
Validation		
Clash score	1.01	5.84
PDB accession code	7RFX	7RFY

Statistics for the highest-resolution shell are shown in parentheses.

Supplementary Table 2. Summary of MERS ORF4b:IMPα2 interactions

Hydrogen Bonds			
#	MERS ORF4b	Dist. (Å)	IMPα2
1	B:ARG 37[NH1]	3.09	A:LEU 104[O]
2	B:ARG 37[NH1]	2.82	A:ARG 106[O]
3	B:ARG 37[NH2]	2.94	A:ARG 106[O]
4	B:ARG 37[NH2]	3.12	A:GLU 107[O]
5	B:ARG 38[O]	3.28	A:TRP 142[NE1]
6	B:ARG 38[N]	3.02	A:ASN 146[OD1]
7	B:ARG 38[O]	2.90	A:ASN 146[ND2]
8	B:ARG 33[NH1]	3.08	A:GLY 150[O]
9	B:ARG 33[NH2]	3.10	A:GLY 150[O]
10	B:ARG 33[NH2]	3.06	A:THR 155[OG1]
11	B:ARG 38[NH1]	2.89	A:GLN 181[OE1]
12	B:LYS 36[O]	3.19	A:TRP 184[NE1]
13	B:LYS 36[N]	2.85	A:ASN 188[OD1]
14	B:LYS 36[O]	2.89	A:ASN 188[ND2]
15	B:TYR 34[N]	3.20	A:GLY 191[O]
16	B:ARG 33[NE]	2.97	A:ASP 192[OD1]
17	B:ARG 33[NH2]	3.21	A:ASP 192[OD1]
18	B:TYR 34[O]	3.09	A:ASN 235[ND2]
19	B:LYS 31[O]	3.10	A:ARG 238[NH1]
20	B:LYS 31[O]	2.82	A:ARG 238[NH2]
21	B:LYS 23[NZ]	2.63	A:VAL 321[O]
22	B:LYS 23[NZ]	2.96	A:THR 328[OG1]
23	B:HIS 26[NE2]	3.16	A:GLU 354[OE2]
24	B:ARG 24[O]	2.86	A:TRP 357[NE1]
25	B:ARG 24[N]	2.74	A:ASN 361[OD1]
26	B:ARG 24[O]	2.93	A:ASN 361[ND2]
27	B:ARG 24[NH2]	3.23	A:GLU 396[OE1]
Salt Bridges			
#	MERS ORF4b	Dist. (Å)	IMPα2
1	B:ARG 33[NE]	2.97	A:ASP 192[OD1]
2	B:HIS 26[NE2]	3.16	A:GLU 354[OE2]
3	B:ARG 24[NH2]	3.23	A:GLU 396[OE1]

Supplementary Table 3. Summary of MERS ORF4b:IMPα3 interactions

Hydrogen Bonds			
#	MERS ORF4b	Dist. (Å)	IMPα3
1	B:ARG 37[NH2]	3.03	A:ASP 102[O]
2	B:ARG 37[NH1]	3.16	A:ASP 102[O]
3	B:ARG 37[NH2]	3.26	A:ASP 102[O]
4	B:ARG 38[O]	3.17	A:TRP 137[NE1]
5	B:ARG 38[N]	2.89	A:ASN 141[OD1]
6	B:ARG 38[O]	3.05	A:ASN 141[ND2]
7	B:ARG 33[NH1]	3.29	A:GLY 145[O]
8	B:ARG 33[NH2]	3.34	A:GLY 145[O]
9	B:ARG 33[NH2]	2.95	A:THR 150[OG1]
10	B:LYS 36[O]	3.19	A:TRP 179[NE1]
11	B:LYS 36[N]	2.95	A:ASN 183[OD1]
12	B:LYS 36[O]	2.92	A:ASN 183[ND2]
13	B:ARG 33[NE]	2.85	A:ASP 187[OD1]
14	B:TYR 34[O]	3.27	A:ASN 226[ND2]
15	B:ARG 24[O]	3.29	A:TRP 348[NE1]
16	B:LYS 23[NZ]	2.83	A:ASN 352[O]
17	B:ARG 24[O]	3.17	A:ASN 352[ND2]
18	B:ARG 24[NH2]	2.67	A:GLU 387[OE1]
Salt Bridges			
#	MERS ORF4b	Dist. (Å)	IMPα3
1	B:ARG 33[NE]	2.85	A:ASP 187[OD1]
2	B:LYS 23[NZ]	3.87	A:ASP 316[OD1]
3	B:HIS 26[NE2]	3.63	A:GLU 345[OE1]
4	B:ARG 24[NH2]	2.67	A:GLU 345[OE1]

Supplementary Table 4. Data collection and refinement statistics.

Data collection and processing	ORF4b WT peptide:IMPα2	ORF4b R24A:IMPα2	ORF4b H26A:IMPα2
Wavelength (Å)	0.9537	0.9537	0.9537
Resolution range (Å)	29.84-1.95 (2.00-1.95)	29.89-1.95 (2.0-1.95)	29.88-1.85 (1.89-1.85)
Space group	P 21 21 21	P 21 21 21	P 21 21 21
Unit cell (Å, ^{o})	78.51 89.53 100.06 90 90 90	78.72 89.68 99.99 90 90 90	78.95 89.65 100.65 90 90 90
Total reflections	681683 (47332)	556716 (19810)	767372 (30173)
Unique reflections	52136 (3622)	52441 (3530)	61555(3637)
Multiplicity	13.1 (13.1)	10.6 (5.6)	12.5 (8.3)
Completeness (%)	100.0 (100.0)	99.8 (97.0)	99.8 (96.9)
Mean I/sigma(I)	14.4 (2.0)	10.5 (1.8)	14.3 (1.7)
Wilson B-factor Å^2	34.65	37.51	29.46
R-merge	0.10 (1.55)	0.11 (0.76)	0.098 (1.34)
R-pim	0.041 (0.64)	0.049 (0.50)	0.041 (0.71)
Refinement			
Number of reflections	52055	47991	61474
Number of R-free reflections	2645	2416	3171
R-work %	18.7	19.4	20.0
R-free %	20.9	21.3	21.17
RMS(bonds)	0.003	0.002	0.002
RMS(angles)	0.60	0.51	0.53
Ramachandran plot			
favored (%)	98.86	98.36	98.86
allowed (%)	1.14	1.64	1.14
outliers (%)	0	0	0
Validation			
Clash score	1.3	0.75	1.01
PDB accession code	7RFZ	7RF0	7RF1

Statistics for the highest-resolution shell are shown in parentheses.

Supplementary Table 5. Data collection and refinement statistics.

Data collection and processing	ORF4b R33A:IMPα2	ORF4b R37A:IMPα2
Wavelength (Å)	0.9537	0.9537
Resolution range (Å)	29.96-2.05 (2.05-2.00)	29.99-2.00 (2.05-2.00)
Space group	P 21 21 21	P 21 21 21
Unit cell (Å, ^{o})	78.85 89.87 100.46 90 90 90	78.73 89.96 100.55 90 90 90
Total reflections	223070 (16974)	357333 (27148)
Unique reflections	48821 (3553)	48968 (3566)
Multiplicity	4.6 (4.8)	7.3 (7.6)
Completeness (%)	99.8 (99.6)	100.0 (100.0)
Mean I/sigma(I)	13.8 (2.0)	15.4 (3.1)
Wilson B-factor Å^2	34.84	32.37
R-merge	0.051 (0.64)	0.064 (0.60)
R-pim	0.040 (0.50)	0.038 (0.35)
Refinement		
Number of reflections	48751	48891
Number of R-free reflections	2440	2442
R-work %	19.8	17.6
R-free %	21.6	19.3
RMS(bonds)	0.002	0.003
RMS(angles)	0.51	0.57
Ramachandran plot		
favored (%)	98.37	98.84
allowed (%)	1.63	1.16
outliers (%)	0	0
Validation		
Clash score	1.48	1.18
PDB accession code	7RF2	7RF3

Statistics for the highest-resolution shell are shown in parentheses.

Supplementary Table 6. Summary of MERS ORF4b WT peptide:IMPα2 interactions

Hydrogen Bonds			
#	ORF4b WT PEPTIDE	Dist. (Å)	IMPα2
1	B:ARG 37[NH1]	3.28	A:LEU 104[O]
2	B:ARG 37[NH1]	2.65	A:ARG 106[O]
3	B:ARG 37[NH2]	3.15	A:ARG 106[O]
4	B:ARG 37[NH2]	3.18	A:GLU 107[O]
5	B:ARG 38[O]	2.99	A:TRP 142[NE1]
6	B:ARG 38[N]	2.91	A:ASN 146[OD1]
7	B:ARG 38[O]	2.83	A:ASN 146[ND2]
8	B:ARG 33[NH1]	2.90	A:GLY 150[O]
9	B:ARG 33[NH2]	2.76	A:GLY 150[O]
10	B:ARG 33[NH2]	3.00	A:THR 155[OG1]
11	B:ARG 38[NH1]	2.66	A:GLN 181[OE1]
12	B:LYS 36[O]	2.90	A:TRP 184[NE1]
13	B:LYS 36[N]	2.78	A:ASN 188[OD1]
14	B:LYS 36[O]	2.92	A:ASN 188[ND2]
15	B:TYR 34[N]	3.33	A:GLY 191[O]
16	B:ARG 33[NE]	2.91	A:ASP 192[OD1]
17	B:LYS 36[NZ]	3.18	A:ASN 228[OD1]
18	B:TYR 34[O]	3.13	A:ASN 235[ND2]
19	B:LYS 31[O]	2.93	A:ARG 238[NH2]
20	B:LYS 23[NZ]	2.68	A:VAL 321[O]
21	B:LYS 23[NZ]	2.67	A:THR 328[OG1]
22	B:HIS 26[NE2]	3.12	A:GLU 354[OE2]
23	B:ARG 24[O]	2.92	A:TRP 357[NE1]
24	B:LYS 23[NZ]	3.30	A:ASN 361[O]
25	B:ARG 24[N]	2.70	A:ASN 361[OD1]
26	B:ARG 24[O]	2.90	A:ASN 361[ND2]
27	B:ARG 24[NH1]	3.10	A:GLU 396[OE1]
28	B:ARG 24[NH2]	3.08	A:GLU 396[OE1]
Salt Bridges			
#	ORF4b WT PEPTIDE	Dist. (Å)	IMPα2
1	B:ARG 33[NE]	2.91	A:ASP 192[OD1]
2	B:HIS 26[NH2]	3.12	A:GLU 354[OE2]
3	B:ARG 24[NH2]	3.08	A:GLU 396[OE1]

Supplementary Table 7. Summary of MERS R24A peptide:IMPα2 interactions

Hydrogen Bonds			
#	ORF4b R24A	Dist. (Å)	IMPα2
1	B:ARG 33[O]	3.04	A:TRP 142[NE1]
2	B:ARG 33[N]	2.95	A:ASN 146[OD1]
3	B:ARG 33[O]	2.98	A:ASN 146[ND2]
4	B:LYS 30[NZ]	2.92	A:GLY 150[O]
5	B:LYS 30[NZ]	2.84	A:THR 155[OG1]
6	B:ARG 33[NH1]	2.47	A:GLN 181[OE1]
7	B:LYS 31[O]	2.96	A:TRP 184[NE1]
8	B:LYS 31[N]	2.92	A:ASN 188[OD1]
9	B:LYS 31[O]	3.02	A:ASN 188[ND2]
10	B:LYS 30[NZ]	3.19	A:ASP 192[OD1]
11	B:LYS 31[NZ]	3.00	A:ASN 228[OD1]
12	B:PRO 28[O]	2.81	A:TRP 231[NE1]
13	B:THR 29[O]	2.86	A:ASN 235[ND2]

Supplementary Table 8. Summary of MERS ORF4b H26A peptide:IMPα2 interactions

Hydrogen Bonds			
#	ORF4b H26A	Dist. (Å)	IMPα2
1	B:ARG 37[NH1]	3.12	A:LEU 104[O]
2	B:ARG 37[NH1]	2.77	A:ARG 106[O]
3	B:ARG 37[NH2]	3.04	A:ARG 106[O]
4	B:ARG 37[NH2]	3.05	A:GLU 107[O]
5	B:ARG 38[O]	3.19	A:TRP 142[NE1]
6	B:ARG 38[N]	3.02	A:ASN 146[OD1]
7	B:ARG 38[O]	2.99	A:ASN 146[ND2]
8	B:ARG 33[NH1]	2.89	A:GLY 150[O]
9	B:ARG 33[NH2]	2.96	A:GLY 150[O]
10	B:ARG 33[NH2]	3.05	A:THR 155[OG1]
11	B:ARG 38[NH1]	2.62	A:GLN 181[OE1]
12	B:LYS 36[O]	3.04	A:TRP 184[NE1]
13	B:LYS 36[N]	2.79	A:ASN 188[OD1]
14	B:LYS 36[O]	2.89	A:ASN 188[ND2]
15	B:TYR 34[N]	3.31	A:GLY 191[O]
16	B:ARG 33[NE]	2.81	A:ASP 192[OD1]
17	B:LYS 36[NH2]	3.13	A:ASN 228[OD1]
18	B:TYR 34[O]	3.14	A:ASN 235[ND2]
19	B:LYS 31[O]	2.87	A:ARG 238[NH2]
20	B:LYS 23[NZ]	2.75	A:VAL 321[O]
21	B:LYS 23[NZ]	2.94	A:THR 328[OG1]
22	B:ARG 24[O]	3.00	A:TRP 357[NE1]
23	B:LYS 23[NZ]	2.88	A:ASN 361[O]
24	B:ARG 24[N]	2.82	A:ASN 361[OD1]
25	B:ARG 24[O]	2.99	A:ASN 361[ND2]
26	B:ARG 24[NH1]	3.29	A:GLU 396[OE1]
27	B:ARG 24[NH2]	3.17	A:GLU 396[OE1]
Salt Bridges			
#	ORF4b H26A	Dist. (Å)	IMPα2
1	B:ARG 33[NE]	2.91	A:ASP 192[OD1]
2	B:ARG 24[NH2]	3.17	A:GLU 396[OE1]

Supplementary Table 9. Summary of MERS ORF4b R33A peptide:IMPα2 interactions

Hydrogen Bonds			
#	ORF4b R33A	Dist. (Å)	IMPα2
1	B:ALA 33[O]	3.05	A:TRP 142[NE1]
2	B:ALA 33[N]	2.81	A:ASN 146[OD1]
3	B:ALA 33[O]	3.04	A:ASN 146[ND2]
4	B:LYS 30[NZ]	3.16	A:GLY 150[O]
5	B:LYS 30[NZ]	2.84	A:THR 155[OG1]
6	B:LYS 31[O]	3.15	A:TRP 184[NE1]
7	B:LYS 31[N]	2.97	A:ASN 188[OD1]
8	B:LYS 31[O]	3.04	A:ASN 188[ND2]
9	B:LYS 30[NZ]	2.95	A:ASP 192[OD1]
10	B:LYS 31[NZ]	3.03	A:ASN 228[OD1]
11	B:THR 29[O]	3.06	A:ASN 235[ND2]
12	B:LYS 23[NZ]	2.69	A:VAL 321[O]
13	B:LYS 23[NZ]	3.05	A:THR 328[OG1]
14	B:ARG 24[O]	2.87	A:TRP 367[NE1]
15	B:ARG 24[N]	2.80	A:ASN 361[OD1]
16	B:ARG 24[O]	2.96	A:ASN 361[ND2]
17	B:ARG 24[NH1]	3.35	A:GLU 396[OE1]
18	B:ARG 24[NH1]	3.26	A:GLU 396[OE1]
Salt Bridges			
#	ORF4b R33A	Dist. (Å)	IMPα2
1	B:LYS 30[NZ]	2.95	A:ASP 192[OD1]
2	B:ARG 24[NH2]	3.26	A:GLU 396[OE1]

Supplementary Table 10. Summary of MERS ORF4b R37A peptide:IMPα2 interactions

Hydrogen Bonds			
#	ORF4b R37A	Dist. (Å)	IMPα2
1	B:LYS 23[NZ]	2.67	A:VAL 321[O]
2	B:LYS 23[NZ]	2.97	A:THR 328[OG1]
3	B:ARG 24[O]	2.95	A:TRP 357[NE1]
4	B:ARG 24[N]	2.79	A:ASN 361[OD1]
5	B:ARG 24[O]	2.86	A:ASN 361[ND2]
6	B:ARG 24[NH1]	3.14	A:GLU 396[OE1]

Supplementary Table 11. Data collection and refinement statistics.

Data collection and processing	NF-kBp50:IMPα2	NF-kBp50:IMPα3	HKU5:IMPα2
Wavelength (Å)	0.9537	0.9537	0.9537
Resolution range (Å)	29.92-2.60 (2.72-2.60)	85.72-2.15 (2.21-2.15)	29.66-2.10 (2.16-2.10)
Space group	P 21 21 21	P 1 21 1	P 21 21 21
Unit cell (Å, °)	78.01 89.75 97.20 90 90 90	47.75 59.18 86.24 90 96.32 90	78.69 90.31 100.00 90 90 90
Total reflections	226419 (26809)	134010 (8703)	209775 (17647)
Unique reflections	21620 (2587)	26271 (2043)	42173 (3428)
Multiplicity	10.5 (10.4)	5.1 (4.3)	5.0 (5.1)
Completeness (%)	99.9 (100.0)	99.6 (95.1)	99.7 (99.9)
Mean I/sigma(I)	10.8 (2.1)	13.4 (2.7)	8.9 (1.6)
Wilson B-factor $Å^2$	55.95	43.14	33.24
R-merge	0.127 (1.321)	0.054 (0.41)	0.104 (1.599)
R-pim	0.059 (0.63)	0.039 (0.31)	0.057 (0.882)
Refinement			
Number of reflections	21554	26150	42042
Number of R-free reflections	1179	1339	2085
R-work %	21.4	22.3	20.4
R-free %	23.9	26.2	22.1
RMS(bonds)	0.002	0.002	0.002
RMS(angles)	0.48	0.49	0.50
Ramachandran plot			
favored (%)	97.67	98.33	98.38
allowed (%)	2.33	1.43	1.62
outliers (%)	0	0.24	0
Validation			
Clash score	1.93	1.36	1.32
PDB accession code	7RF4	7RF5	7RF6

Statistics for the highest-resolution shell are shown in parentheses.

Supplementary Table 12. Summary of IMPα2:NF-kB p50 interactions

Hydrogen Bonds			
#	NF-kB	Dist. (Å)	IMPα2
1	B:MET 367[SD]	3.29	E:ARG 101[NH1]
2	B:GLN 364[NE2]	2.78	E:LEU 104[O]
3	B:LYS 365[O]	3.31	E:TRP 142[NE1]
4	B:LYS 365[N]	2.89	E:ASN 146[OD1]
5	B:LYS 365[O]	3.00	E:ASN 146[ND2]
6	B:LYS 362[NZ]	2.73	E:GLY 150[O]
7	B:LYS 362[NZ]	3.09	E:THR 155[OG1]
8	B:LYS 365[NZ]	2.68	E:GLN 181[OE1]
9	B:ARG 363[O]	2.86	E:TRP 184[NE1]
10	B:ARG 363[N]	2.80	E:ASN 188[OD1]
11	B:ARG 363[O]	3.03	E:ASN 188[ND2]
12	B:LYS 362[NZ]	3.23	E:ASP 192[OD1]
13	B:ARG 363[NH2]	3.07	E:ASN 228[OD1]
14	B:ARG 361[O]	3.00	E:ASN 235[ND2]

Supplementary Table 13. Summary of IMPα3:NF-kB p50 interactions

Hydrogen Bonds			
#	NF-kB	Dist. (Å)	IMPα3
1	B:GLN 364[NE2]	3.12	A:LEU 99[O]
2	B:MET 367[N]	3.04	A:SER 100[O]
3	B:GLN 364[NE2]	2.92	A:ASP 102[O]
4	B:LYS 365[O]	2.92	A:TRP 137[NE1]
5	B:LYS 365[N]	2.82	A:ASN 141[OD1]
6	B:LYS 365[O]	2.99	A:ASN 141[ND2]
7	B:LYS 362[NZ]	2.89	A:GLY 145[O]
8	B:LYS 362[NZ]	2.77	A:THR 150[OG1]
9	B:ARG 363[O]	3.05	A:TRP 179[NE1]
10	B:ARG 363[N]	2.78	A:ASN 183[OD1]
11	B:ARG 363[O]	2.79	A:ASN 183[ND2]
12	B:ARG 361[NH1]	2.50	A:GLY 186[O]
13	B:ARG 361[O]	3.07	A:TRP 222[NE1]
14	B:ARG 361[O]	2.71	A:ASN 226[ND2]

Supplementary Table 14. Summary of HKU5:IMPα2 interactions

Hydrogen Bonds			
#	HKU5	Dist. (Å)	IMPα2
1	C:ARG 34[NH1]	3.07	A:LEU 104[O]
2	C:ARG 34[NH1]	2.71	A:ARG 106[O]
3	C:ARG 34[NH2]	3.03	A:ARG 106[O]
4	C:ARG 34[NH2]	3.06	A:GLU 107[O]
5	C:ARG 35[O]	3.25	A:TRP 142[NE1]
6	C:ARG 35[N]	3.24	A:ASN 146[OD1]
7	C:ARG 35[O]	3.15	A:ASN 146[ND2]
8	C:LYS 32[NZ]	2.90	A:GLY 150[O]
9	C:LYS 32[NZ]	2.80	A:THR 155[OG1]
10	C:ARG 35[NH1]	2.61	A:GLN 181[OE1]
11	C:ARG 33[O]	2.86	A:TRP 184[NE1]
12	C:ARG 33[N]	2.85	A:ASN 188[OD1]
13	C:ARG 33[O]	2.98	A:ASN 188[ND2]
14	C:LYS 32[NZ]	3.28	A:ASP 192[OD1]
15	C:ARG 33[NH1]	2.88	A:ASN 228[OD1]
16	C:ARG 31[O]	3.07	A:ASN 235[ND2]

References

1 The PyMOL Molecular Graphic System v. 2.5.0 (2010).

2 Hoad, M., Forwood, J. *PDB 7L04: Crystal Structure of Adeno-Associated Virus Porcine Origin capsid protein in complex with Importin-alpha 2*, <https://www.rcsb.org/structure/7L04> (2021).

3 Tay, M. Y. *et al.* The C-terminal 18 Amino Acid Region of Dengue Virus NS5 Regulates its Subcellular Localization and Contains a Conserved Arginine Residue Essential for Infectious Virus Production. *PLoS Pathog* **12**, e1005886, doi:10.1371/journal.ppat.1005886 (2016).

4 Sankhala, R. S., Lokareddy, R. K. & Cingolani, G. Divergent Evolution of Nuclear Localization Signal Sequences in Herpesvirus Terminase Subunits. *J Biol Chem* **291**, 11420-11433, doi:10.1074/jbc.M116.724393 (2016).

5 Smith, K. M., Himiari, Z., Tsimbalyuk, S. & Forwood, J. K. Structural Basis for Importin-alpha Binding of the Human Immunodeficiency Virus Tat. *Sci Rep* **7**, 1650, doi:10.1038/s41598-017-01853-7 (2017).

6 Ng, I. H. W. *et al.* Zika Virus NS5 Forms Supramolecular Nuclear Bodies That Sequester Importin-alpha and Modulate the Host Immune and Pro-Inflammatory Response in Neuronal Cells. *ACS Infect Dis* **5**, 932-948, doi:10.1021/acsinfecdis.8b00373 (2019).

7 Nakada, R., Hirano, H. & Matsuura, Y. Structural basis for the regulation of nuclear import of Epstein-Barr virus nuclear antigen 1 (EBNA1) by phosphorylation of the nuclear localization signal. *Biochem Biophys Res Commun* **484**, 113-117, doi:10.1016/j.bbrc.2017.01.063 (2017).

8 Smith, K. M. *et al.* Structural basis for importin alpha 3 specificity of W proteins in Hendra and Nipah viruses. *Nat Commun* **9**, 3703, doi:10.1038/s41467-018-05928-5 (2018).

9 Nakada, R. & Matsuura, Y. Crystal structure of importin-alpha bound to the nuclear localization signal of Epstein-Barr virus EBNA-LP protein. *Protein Sci* **26**, 1231-1235, doi:10.1002/pro.3173 (2017).
