# Supplementary material for: MERS-CoV ORF4b employs an unusual binding mechanism to target IMPα and block innate immunity
Source: Nat Commun. 2022 Mar 25;13:1604. doi: 10.1038/s41467-022-28851-2 (PMC8956657; doi:10.1038/s41467-022-28851-2)
Supplement: Supplementary file 2 — Reporting Summary [file 41467_2022_28851_MOESM2_ESM.pdf]

nature portfolio

Corresponding author(s): Jade Forwood

Last updated by author(s): Sep 4, 2021

Reporting Summary

Nature Portfolio wishes to improve the reproducibility of the work that we publish. This form provides structure for consistency and transparency in reporting. For further information on Nature Portfolio policies, see our Editorial Policies and the Editorial Policy Checklist.

Statistics

For all statistical analyses, confirm that the following items are present in the figure legend, table legend, main text, or Methods section.

n/a	Confirmed	
☐	☒	The exact sample size (n) for each experimental group/condition, given as a discrete number and unit of measurement
☐	☒	A statement on whether measurements were taken from distinct samples or whether the same sample was measured repeatedly
☐	☒	The statistical test(s) used AND whether they are one- or two-sided<br>*Only common tests should be described solely by name; describe more complex techniques in the Methods section.*
☒	☐	A description of all covariates tested
☒	☐	A description of any assumptions or corrections, such as tests of normality and adjustment for multiple comparisons
☒	☐	A full description of the statistical parameters including central tendency (e.g. means) or other basic estimates (e.g. regression coefficient) AND variation (e.g. standard deviation) or associated estimates of uncertainty (e.g. confidence intervals)
☒	☐	For null hypothesis testing, the test statistic (e.g. F, t, r) with confidence intervals, effect sizes, degrees of freedom and P value noted<br>*Give P values as exact values whenever suitable.*
☒	☐	For Bayesian analysis, information on the choice of priors and Markov chain Monte Carlo settings
☒	☐	For hierarchical and complex designs, identification of the appropriate level for tests and full reporting of outcomes
☒	☐	Estimates of effect sizes (e.g. Cohen's d, Pearson's r), indicating how they were calculated

Our web collection on statistics for biologists contains articles on many of the points above.

Software and code

Policy information about availability of computer code

Data collection	No software was used
Data analysis	No software was used

For manuscripts utilizing custom algorithms or software that are central to the research but not yet described in published literature, software must be made available to editors and reviewers. We strongly encourage code deposition in a community repository (e.g. GitHub). See the Nature Portfolio guidelines for submitting code & software for further information.

Data

Policy information about availability of data

All manuscripts must include a data availability statement. This statement should provide the following information, where applicable:

- Accession codes, unique identifiers, or web links for publicly available datasets
- A description of any restrictions on data availability
- For clinical datasets or third party data, please ensure that the statement adheres to our policy

7RFX 7RFY 7RFZ 7RF0 7RF1 7RF2 7RF3 7RF4 7RF5 7RF6

Field-specific reporting

Life sciences study design

All studies must disclose on these points even when the disclosure is negative.

Sample size	Sample size analysis was not required in this study
Data exclusions	No data was excluded
Replication	All attempts at replication were successul
Randomization	Not relevant. The experiments did not require randomization
Blinding	The experiments did not require blinding

Reporting for specific materials, systems and methods

We require information from authors about some types of materials, experimental systems and methods used in many studies. Here, indicate whether each material, system or method listed is relevant to your study. If you are not sure if a list item applies to your research, read the appropriate section before selecting a response.

Materials & experimental systems

n/a	Involved in the study	
☐	☒	Antibodies
☐	☒	Eukaryotic cell lines
☒	☐	Palaeontology and archaeology
☒	☐	Animals and other organisms
☒	☐	Human research participants
☒	☐	Clinical data
☒	☐	Dual use research of concern

Methods

n/a	Involved in the study	
☒	☐	ChIP-seq
☒	☐	Flow cytometry
☒	☐	MRI-based neuroimaging

Antibodies

Antibodies used

Antibodies – lot numbers have been included where available
Primary Antibodies
anti-DYKDDDK tag monoclonal antibody conjugated to Alexa Fluor 488 (Invitrogen, cat# MA1-142-A488, lot# UB276601)
Validation: Invitrogen states that 'This Antibody was verified by Relative expression to ensure that the antibody binds to the antigen stated. Antibody specificity was demonstrated by detection of different targets fused to DYKDDDDK tag in transiently transfected lysates tested.'

anti-p65 (Santa Cruz, cat# sc-109, lot# F2304)
Validation: Santa Cruz states that the epitope the antibody reacts too was mapped within the N-terminus of NFκB p65 of human origin.

rabbit anti-Flag (Sigma Aldrich, cat# F7425)
Validation: Sigma Aldrich states that 'The rabbit Anti-FLAG polyclonal affinity antibody ANTI-FLAG recognizes the FLAG epitope located on FLAG fusion proteins. This antibody reacts with N-terminal, N-terminal-Met, and C-terminal FLAG fusion proteins.'

mouse anti--tubulin (Sigma Aldrich, cat# T8328)
Validation: Sigma Aldrich states that the 'Mouse anti-β-tubulin antibody reacts specifically with β tubulin, types I, II, III, and IV of bovine, rat, mouse and human.'

Rabbit anti-HA (SG77) (Invitrogen, cat# 71-5500)
Validation: Invitrogen states that the antibody was generated to and reacts to the HA-tag YPYDVPDYA.

Mouse anti-GAPDH (GA1R) (Invitrogen, cat# MA5-15738)
Validation: Antibody was generated to recombinant GAPDH. Invitrogen states that 'This antibody detects GAPDH from BL-21 bacteria, Sf9 insect, Saccharomyces cerevisiae (yeast), human, mouse, rat, rabbit, hamster, and chicken samples.'

Secondary Antibodies
goat anti-rabbit IgG Alexa Fluor 647 (Invitrogen, cat# A-21244, lot# 1871168)

Anti-rabbit IgG, HRP-linked antibody #7074 (Cell Signaling Technology, cat# 7074)

Anti-mouse IgG, HRP-linked antibody #7076 (Cell Signaling Technology, cat# 7076)

Validation	Antibodies – lot numbers have been included where available<br>Primary Antibodies<br>anti-DYKDDDK tag monoclonal antibody conjugated to Alexa Fluor 488 (Invitrogen, cat# MA1-142-A488, lot# UB276601)<br>Validation: Invitrogen states that 'This Antibody was verified by Relative expression to ensure that the antibody binds to the antigen stated. Antibody specificity was demonstrated by detection of different targets fused to DYKDDDDK tag in transiently transfected lysates tested.'<br><br>anti-p65 (Santa Cruz, cat# sc-109, lot# F2304)<br>Validation: Santa Cruz states that the epitope the antibody reacts too was mapped within the N-terminus of NFκB p65 of human origin.<br><br>rabbit anti-Flag (Sigma Aldrich, cat# F7425)<br>Validation: Sigma Aldrich states that 'The rabbit Anti-FLAG polyclonal affinity antibody ANTI-FLAG recognizes the FLAG epitope located on FLAG fusion proteins. This antibody reacts with N-terminal, N-terminal-Met, and C-terminal FLAG fusion proteins.'<br><br>mouse anti--tubulin (Sigma Aldrich, cat# T8328)<br>Validation: Sigma Aldrich states that the 'Mouse anti-β-tubulin antibody reacts specifically with β tubulin, types I, II, III, and IV of bovine, rat, mouse and human.'<br><br>Rabbit anti-HA (SG77) (Invitrogen, cat# 71-5500)<br>Validation: Invitrogen states that the antibody was generated to and reacts to the HA-tag YPYDVPDYA.<br><br>Mouse anti-GAPDH (GA1R) (Invitrogen, cat# MA5-15738)<br>Validation: Antibody was generated to recombinant GAPDH. Invitrogen states that 'This antibody detects GAPDH from BL-21 bacteria, Sf9 insect, Saccharomyces cerevisiae (yeast), human, mouse, rat, rabbit, hamster, and chicken samples.'<br><br>Secondary Antibodies<br>goat anti-rabbit IgG Alexa Fluor 647 (Invitrogen, cat# A-21244, lot# 1871168)<br><br>Anti-rabbit IgG, HRP-linked antibody #7074 (Cell Signaling Technology, cat# 7074)<br><br>Anti-mouse IgG, HRP-linked antibody #7076 (Cell Signaling Technology, cat# 7076)

Eukaryotic cell lines

Policy information about cell lines

Cell line source(s)	HEK293T cells (CRL-3216) were obtained from ATCC. Huh7 cells were a generous gift from the Gordan lab at University of California at San Francisco.
Authentication	We monitor cells to confirm that their morphology and growth properties are consistent with the originally purchased cells. If changes are noted, the cells are discarded and we return to an early passage that has been cryopreserved
Mycoplasma contamination	HEK293T and Huh7 cells are mycoplasma free, and are frequently tested using the MycoAlert™ PLUS Mycoplasma Detection Kit (Lonza, cat# LT07-703)
Commonly misidentified lines<br>(See ICLAC register)	*Name any commonly misidentified cell lines used in the study and provide a rationale for their use.*
